# Advanced image reconstruction algorithms for high-resolution digital time-of-flight PET/CT enhance visualization of sub-clinical internal mammary lymph node metastases in breast cancer: a phantom and a clinical, retrospective cohort study

**DOI:** 10.1186/s40658-026-00846-8

**Published:** 2026-02-22

**Authors:** Yoko Satoh, Kenta Miwa, Akinori Takenaka, Yoshitaka Inui, Masanori Watanabe, Tensho Yamao, Noriaki Miyaji, Seiichiro Ota, Edwin K. Leung, Xibin Quan, Hiroshi Toyama, Masanori Inoue

**Affiliations:** 1 Imaging Center, Fujita Medical Innovation Center Tokyo, 1-1-4 Hanedakuko, Ota-ku, Tokyo, Japan; 2 Department of Radiological Sciences, School of Health Sciences, Fukushima Medical University, Fukushima, Japan; 3 Department of Radiology, Fujita Health University School of Medicine, Aichi, Japan; 4 Department of Radiology, Fujita Health University Hospital, Aichi, Japan; 5 United Imaging Healthcare North America, Inc., Houston, TX USA; 6 Shanghai United Imaging Healthcare Co. Ltd., Shanghai, China

**Keywords:** Digital PET/CT, Image reconstruction, Intramammary lymph node metastasis, Breast cancer, Diagnostic performance

## Abstract

**Background:**

Internal mammary lymph node (IMLN) metastases play an important role in breast cancer staging and treatment planning but is often difficult to detect because of their small size and anatomical location. Recent advances in digital time-of-flight (TOF) positron emission tomography (PET)/CT and advanced image reconstruction techniques may improve the visualization of such small lesions. This study aimed to evaluate the performance of advanced reconstruction methods (HYPER Iterative and uAI HYPER DPR) for visualizing IMLN metastases in breast cancer using phantom and clinical data.

**Methods:**

A modified NEMA image quality phantom and a retrospective cohort of breast cancer patients with IMLN metastases were evaluated using a high-resolution digital TOF PET/CT system (uMI 550). Images were reconstructed using ordered subset expectation maximization (OSEM), HYPER Iterative, and uAI HYPER DPR with different reconstruction parameters, and quantitative metrics and visual scores were assessed.

**Results:**

In both phantom and clinical images, smaller RS-values for HYPER Iterative and larger Str-values for uAI HYPER DPR were associated with higher lesion conspicuity and contrast-related metrics, at the expense of increased noise. Images reconstructed with a 256 × 256 matrix showed lower background variability than those reconstructed with a 512 × 512 matrix. In the clinical study, these reconstruction settings resulted in higher SUV_max_ and tumor-to-background ratios for IMLN metastases, and visual scores for diagnostic confidence were higher for HYPER Iterative (RS = 0.7–0.91) and uAI HYPER DPR (Str = 2–4) than for OSEM.

**Supplementary Information:**

The online version contains supplementary material available at 10.1186/s40658-026-00846-8.

## Background

^18^F-fluorodeoxyglucose (FDG) positron emission tomography/computed tomography (PET/CT) is an essential imaging modality for staging and re-staging, monitoring treatment response to therapy, and predicting the prognosis of patients with breast cancer (BC) [1–3]. FDG PET is particularly valuable for evaluating lymph node metastasis in BC staging, especially for detecting internal mammary lymph node (IMLN) metastasis [4–6]. Unlike axillary or supraclavicular lymph node metastases, IMLN metastases are less likely to be identified by ultrasound or contrast-enhanced CT due to their smaller size and location within the chest wall. The presence of IMLN metastasis leads to up-staging of BC, influencing treatment decisions and recurrence risk [7]. This is especially crucial for BC patients undergoing neoadjuvant chemotherapy, where imaging determines the final stage, highlighting the importance of accurate scoring using FDG PET.

Recent advancements in PET/CT technology have improved lesion detectability, particularly for small and low-uptake lesions [8]. These developments include improved time-of-flight (TOF) performance enabled by modern scintillator materials, digital and silicon photomultiplier (SiPM)-based detectors, smaller scintillation crystals for enhanced spatial resolution, and the emergence of long axial field-of-view (FOV) PET systems. While these innovations have collectively raised the performance ceiling of PET/CT imaging, key system characteristics – including TOF resolution, spatial resolution, and system sensitivity – remain strongly dependent on detector design and overall system architecture and therefore vary across PET/CT platforms and vendors [9] .

Beyond hardware improvements, substantial gains in PET image quality have also been made possible through advances in image reconstruction. Advanced reconstruction approaches have enabled improved trade-offs between image noise, contrast recovery, and spatial resolution. Among these, Bayesian penalized likelihood (BPL) algorithms and, more recently, deep learning-based reconstruction methods have been introduced to enhance lesion conspicuity and quantitative performance [10–12].

Despite the growing availability of advanced reconstruction techniques, their impact on clinically relevant tasks–such as the detection and quantification of small or low-contrast lesions–remains an area of active investigation. In particular, the extent to which differences between reconstruction approaches translate into meaningful improvements in lesion conspicuity, quantitative accuracy, and diagnostic confidence under routine clinical acquisition conditions is not yet fully established. This uncertainty provides the motivation for the present study, which evaluates the influence of different reconstruction strategies on the assessment of internal mammary lymph node (IMLN) metastases.

A common method for reconstructing PET images is to utilize the TOF-enabled ordered subset expectation maximization (OSEM) algorithm [13]. HYPER Iterative and uAI HYPER deep progressive reconstruction (DPR) are two advanced image reconstruction algorithms that have been developed for high-resolution digital TOF PET/CT scanners. HYPER Iterative is based on a Bayesian estimation likelihood algorithm that utilizes a total variation regularized expectation maximization (TVREM) method with a penalty term, which incorporates the total variation between pixels, the global noise equivalent count, and the local sensitivity profile [14, 15]. The penalization factor (or regularization strength) in HYPER Iterative controls the tradeoff between noise level and resolution of the image. It has previously been shown that HYPER Iterative produced good image quality and diagnostic performance for patients with a wide range of body mass index in ultra-low dose ^18^F-FDG PET/CT examinations [16]. On the other hand, uAI HYPER DPR integrates convolutional neural networks (CNNs) that have been pre-trained on true signals and noise into the OSEM algorithm [17–19]. It is designed to improve image sharpness, reduce noise, and enhance the visualization of small structures or lesions, while maintaining accurate quantitative measurements.

To the best of our knowledge, there is no study that has evaluated the optimal reconstruction parameters for HYPER Iterative and uAI HYPER DPR for visualizing IMLN metastases. Therefore, the purpose of this study was to evaluate how these advanced image reconstruction methods can best leverage high-resolution digital TOF PET/CT to detect small, clinically significant pre-clinical IMLN metastases in BC using phantom and clinical datasets.

## Methods

### PET/CT scanner

All data were acquired using a uMI 550 high-resolution digital TOF PET/CT system (United Imaging Healthcare, Shanghai, China). The system is comprised of a PET scanner coupled to an 80-slice CT scanner. One detector block of the PET scanner is comprised of a 7 × 6 LYSO array of 2.76 × 2.76 × 16.3 mm^3^ crystals coupled to SiPM sensors. The uMI 550 has axial and transaxial FOV of 24 and 70 cm, respectively. The TOF resolution is 372 ps. The spatial resolution and sensitivity of the uMI 550 according to the National Electrical Manufacturers Association (NEMA) NU 2-2018 standard are 2.95 mm/2.97 mm (transverse/axial) at 10 mm radial offset and 10.24 cps/kBq, respectively [20].

### Phantom design

**Fig. 1:**
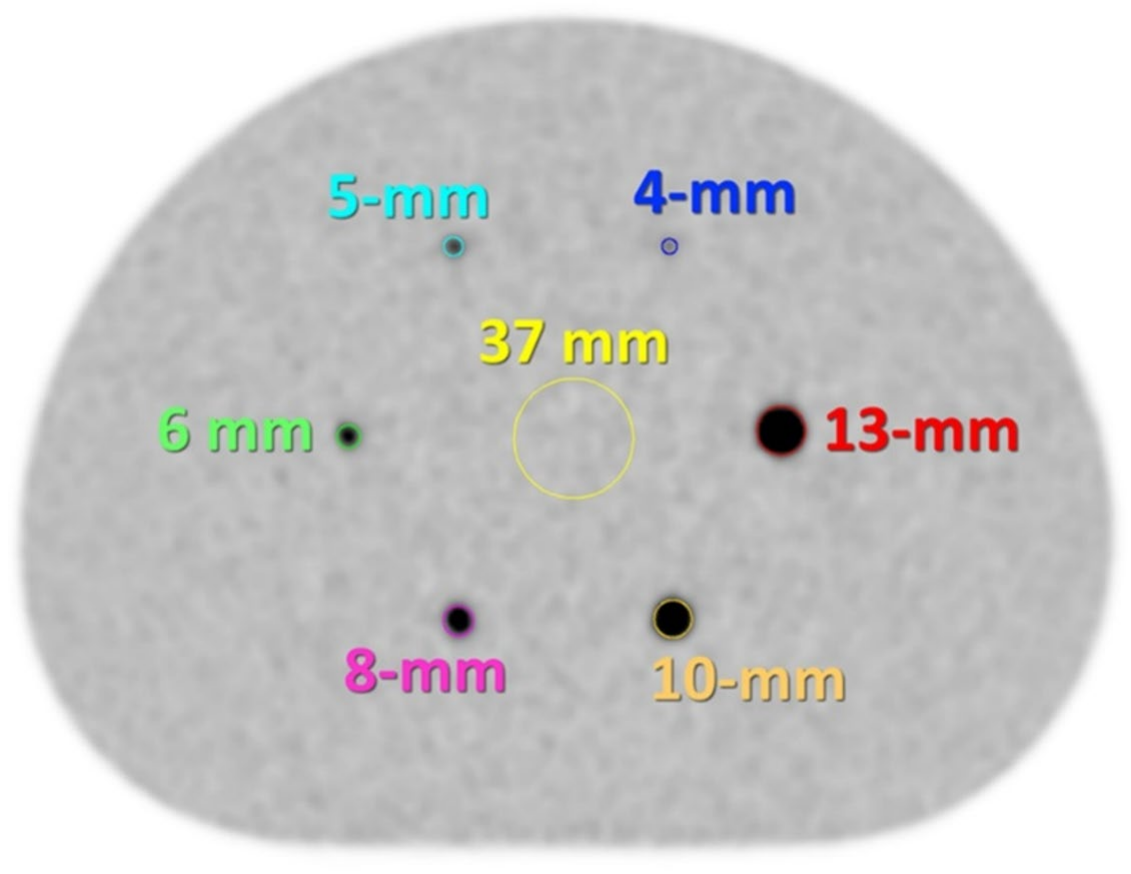
An axial PET image of the modified NEMA IQ phantom containing six smaller spheres (4-, 5-, 6-, 8-, 10-, and 13-mm in diameter) with corresponding regions of interest (ROIs) that match the sphere diameters, centered based on the CT images. A ROI of 37-mm in diameter was placed on the center of the phantom for background measurement

A modified NEMA image quality (IQ) phantom based on the NEMA NU 2-2018 standard was used in this study. The standard NEMA NU 2-2018 IQ phantom consists of 6 spheres with diameters ranging from 10- to 37-mm. In conventional morphological imaging such as CT and MRI, a short-axis diameter of 10-mm has traditionally been used as a criterion for suspected lymph node metastasis. However, FDG PET frequently identifies metastatic lymph nodes with short-axis diameters of less than 10-mm. Accordingly, evaluating the detectability of sub-centimeter lymph node metastases is clinically important, particularly when using whole-body PET/CT systems. Accordingly, a modified NEMA IQ phantom containing smaller spheres (with diameters of 4-, 5-, 6-, 8-, 10-, and 13-mm) was filled with ^18^F-FDG (Fig. 1). These sphere sizes were selected to cover sub-centimeter lesions of clinical significance, with the minimum detectable lesion size assuming 4 mm. The sphere-to-background radioactivity concentration ratio in the phantom was 8:1, in contrast to the 4:1 ratio commonly used in phantom studies for whole-body PET imaging, to reflect clinically realistic FDG uptake conditions for internal mammary lymph node metastases, which are typically surrounded by tissues with low physiological FDG uptake, such as the ribs, sternum, and intercostal muscles. The background activity concentration of 5.3 kBq/mL chosen based on the previous report [10]. All phantom acquisitions were performed under standardized conditions following the Japanese Society of Nuclear Medicine recommendations, using clinically relevant acquisition times and reconstruction settings consistent with routine whole-body PET/CT imaging [21].

### Patient selection

The retrospective study was approved by our institutional review board (HM24-351), which waived the requirement for written informed consent. Patients were included based on the following criteria: (1) pre-treatment BC patients who underwent ^18^F-FDG PET/CT between June 2020 and May 2024, (2) no history of malignancy, and (3) the presence of ipsilateral IMLN metastases with a diameter of 1 cm or less. The diagnosis of IMLN metastasis was established by either (1) evidence of suspected metastasis identified on two or more imaging modalities—PET/CT, contrast-enhanced CT, contrast-enhanced MRI, or breast ultrasound—with a confirmed reduction in lesion size following chemotherapy, or (2) a diagnosis determined by a multidisciplinary cancer board consisting of breast surgeons and radiologists, even if the patient did not meet the first criterion.

### Data acquisition and image reconstruction

For the NEMA IQ phantom scan, data were acquired in a single acquisition for 30 min, and re-binned with a 2-min acquisition time. In our clinical protocol, the acquisition time per bed position was set to 2.5 min, which was determined during system installation by balancing count statistics with clinical throughput and patient tolerability. In phantom studies, slightly shorter acquisition time was employed intentionally. This is because the phantom lacks physiological activity other than the hot sphere and background, resulting in reduced scattering and a more controlled imaging environment. Therefore, a 2-min re-binning was a reasonable setting for evaluating reconstruction performance in severe counting conditions. Images were then reconstructed using three algorithms: (1) TOF-enabled OSEM with point spread function (PSF), with 3 iterations and 20 subsets, and edge-preserving non-local mean (NLM) and 6-mm Gaussian post-reconstruction filters. The reconstruction was based on a vendor-specific preset (“Smooth3”), for which the default Gaussian smoothing is 3-mm; however, a 6-mm Gaussian filter was selected during clinical implementation to achieve an appropriate balance between image contrast and noise under routine whole-body PET/CT conditions, (2) HYPER Iterative (with β-values ranging from 0.01 to 0.98), and (3) uAI HYPER DPR (with smoothing strength from 1 to 5 using a vendor-specific preset ranging from “Smooth” to “Sharp”), both reconstructed without additional post-reconstruction filtering. In HYPER Iterative reconstruction, the regularization strength can be expressed either as the penalization factor β or as the relative strength (RS), depending on the software interface. RS represents a normalized form of the effective regularization strength derived from β and was used in this study for parameter labeling, as it facilitates intuitive comparison across reconstruction settings. Lower RS values are generally associated with higher image sharpness and increased noise, whereas higher RS values result in smoother images with reduced noise. In uAI HYPER DPR, the reconstruction strength is controlled by the parameter Str, which reflects the balance between noise suppression and edge preservation. Higher Str values emphasize clearer images with higher noise, while lower Str values lead to increased smoothing and noise reduction. For the phantom study, a low-dose CT scan was performed for attenuation correction and anatomical reference using the standard clinical PET/CT protocol at our institution.

For the clinical scans, the patients were fasted for at least 6 h before the administration of ^18^F-FDG (at 3.7 MBq/kg), and scanned in the supine position for 2.5 min per bed position, starting at 1 h post-injection. For patient studies, CT was acquired using a routine low-dose protocol for attenuation correction and anatomical localization, in accordance with standard clinical PET/CT practice.

All images were reconstructed using matrix sizes of 256 × 256 and 512 × 512 (corresponding to pixel sizes of 2.344 mm and 1.172 mm, respectively) with a slice thickness of 2 mm to evaluate the effects of using smaller voxels for visualizing smaller lesions. The slice thickness was kept constant at 2 mm for both matrix sizes to isolate the effect of in-plane voxel size on lesion visualization and quantitative metrics.

### Image quality analysis

The phantom images were analyzed using PMOD Ver. 4.0 (PMOD Technologies LLC, Zurich, Switzerland). As shown in Fig. 1, circular ROIs matching the diameter of the hot spheres were placed on the central slice of each hot sphere, using CT image guidance. In addition, a ROI of 37-mm in diameter was placed on the center of the phantom for background measurement. Three quantitative PET parameters for evaluating image quality were selected based on a previous study on image reconstruction for dedicated breast PET [22]. The coefficient of variation of the background ($\:{CV}_{BG}$), detectability index ($\:DI$) of the smallest sphere that can be visually detected, and recovery coefficient ($\:RC$) were calculated according to the following equations:

$$\:{CV}_{BG}\:=\:\frac{{SD}_{BG}}{{C}_{BG;mean}}$$

$$\:DI\:=({C}_{H;max}-{C}_{BG;mean})/{SD}_{BG}$$

$$\:RC\:={C}_{H;max}/{C}_{BG;mean}$$

where $\:{SD}_{BG}$ and $\:{C}_{BG;mean}$ are the standard deviation and mean of standardized uptake value (SUV) of the background ROI. $\:{C}_{H;max}$ is the SUV_max_ of the smallest sphere (4-mm) that can be visually detected. Because the phantom spheres were very small and often visualized on only one or two axial slices, mean-based measurements were considered unreliable; therefore, maximum-based metrics (DI and RC) were adopted for quantitative evaluation.

**Fig. 2:**
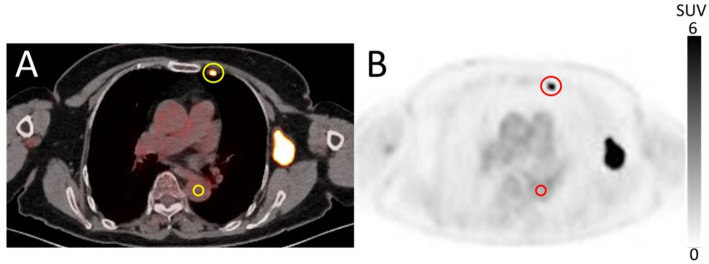
Representative VOI placements on the clinical **A** PET/CT and **B** PET images. A VOI of sufficiently large size was placed on the metastatic IMLN tumor, and a VOI of 1.5 cm in diameter was placed in the descending aorta (as the background)

For the quantitative evaluation of the clinical images, the SUV_max_ of IMLN metastasis, contrast (SUV_max_ of IMLN metastasis / SUV_mean_ of background placed in the descending aorta at the same slice position as the IMLN for evaluation), and $\:{CV}_{BG}$ were calculated (Fig. 2). The clinical datasets were analyzed using a modified, in-house version of Metavol [23].

For the qualitative evaluation of the clinical images, one of the four experienced nuclear medicine physicians randomly presented the transverse PET/CT fusion and PET images paired with IMLN metastasis to the other three readers. The readers independently assessed all PET/CT images while blinded to the reconstruction settings and clinical background. The images were scored according to the confidence of IMLN metastasis diagnosis and image noise level, using a three-point scale (ranging between a score of 0 [low], 1 [medium], and 2 [high]). The final score was the average score of the three readers. The inter-reader agreement was evaluated by calculating Cohen’s Kappa using JMP Pro 17 (17.2.0) software (SAS Institute Japan, Tokyo, Japan). This retrospective analysis focused on the comparison of reconstruction methods and parameter settings; the impact of reconstruction-related differences on actual clinical management was not evaluated.

## Results

### Phantom evaluation

**Fig. 3:**
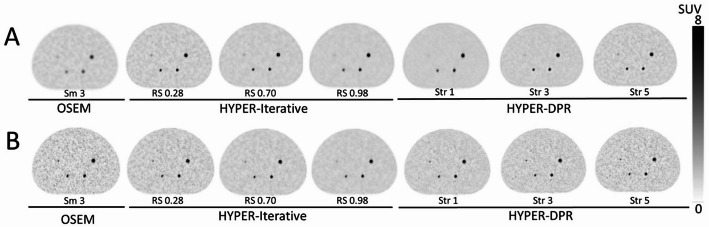
Representative modified NEMA Image Quality (IQ) phantom images reconstructed using OSEM, HYPER Iterative, and uAI HYPER DPR. The matrix sizes used were **A** 256 × 256 and **B** 512 × 512, with a slice thickness of 2 mm. The reconstruction settings are indicated below each image (OSEM: Smooth 3; HYPER Iterative: RS values; uAI HYPER DPR: Str values)

The PET images of the modified NEMA IQ phantom reconstructed using OSEM, HYPER Iterative, and uAI HYPER DPR are shown in Fig. 3. The hotspots were more visible in images with a matrix size of 512 × 512 than in those with 256 × 256, and they were more clearly visualized on both the HYPER Iterative and uAI HYPER DPR images than those of OSEM. Spheres that were 6-mm or greater in diameter were visible for all reconstructed images, while the 4-mm and 5-mm spheres were not visible in all reconstructed settings evaluated. Therefore, only the values for the smallest visible sphere (6-mm in diameter) for all reconstructed images were used to calculate $\:DI$ and $\:RC$.

**Fig. 4:**
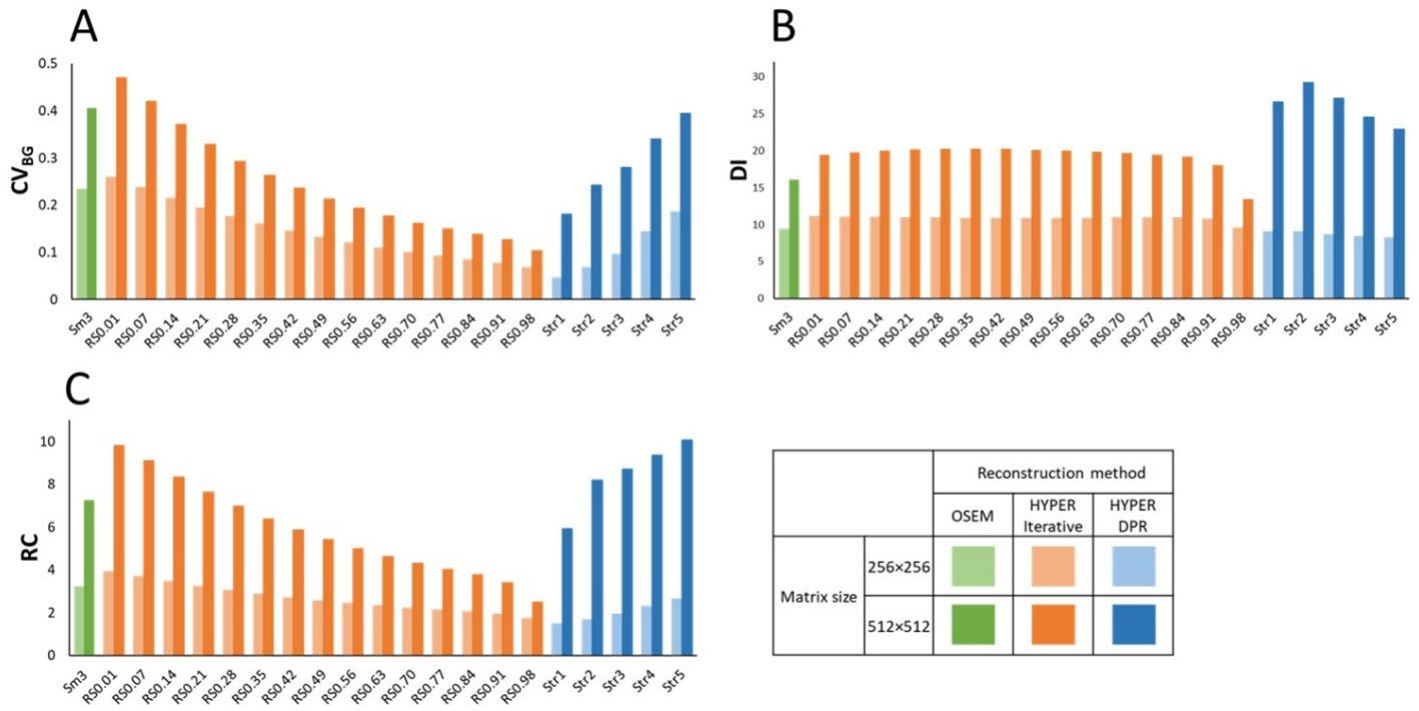
Quantitative phantom evaluation results. **A** Coefficient of variation of the background ($\:{CV}_{BG}$), **B** detectability index ($\:DI$), and **C** recovery coefficient ($\:RC$) of the phantom images reconstructed using OSEM, HYPER Iterative, and uAI HYPER DPR. Results for both matrix sizes (256 × 256 and 512 × 512) are shown. The reconstruction settings for each method are indicated on the x-axis (OSEM: Smooth 3; HYPER Iterative: RS values; uAI HYPER DPR: Str values)

In the quantitative evaluation, $\:{CV}_{BG}$, $\:DI$, and $\:RC$ were higher in images with a matrix size of 512 × 512 than those with 256 × 256 for all image reconstruction methods (Fig. 4). For clarity, representative quantitative results are reported here for the 512 × 512 matrix. With a 512 × 512 matrix, CV_BG_ for OSEM was 0.41, whereas it ranged from 0.10 to 0.47 for HYPER Iterative and from 0.18 to 0.39 for uAI HYPER DPR, depending on the RS or Str values (Fig. 4A). For the smallest visible sphere (6-mm in diameter) with a 512 × 512 matrix, DI for OSEM was 16.1, whereas DI ranged from 13 0.4 to 20.3 for HYPER Iterative and from 19.4 to 20.3 for uAI HYPER DPR (Fig. 4B). Similarly, RC for the 6-mm sphere was 16.1 for OSEM, and ranged from 13.4 to 20.3 for HYPER Iterative and from 22.9 to 29.2 for uAI HYPER DPR (Fig. 4C).

Selecting smaller RS for HYPER Iterative and larger Str for uAI HYPER DPR resulted in higher $\:{CV}_{BG}$. When using a matrix size 512 × 512, $\:DI$ was highest in uAI HYPER DPR, followed by HYPER Iterative. Notably, *DI* for HYPER Iterative showed a relatively narrow range across different RS values for both 256 × 256 and 512 × 512 matrices, as shown in Fig. 4B.

Based on the visual balance between the background noise and the clarity of the spheres in the phantom study, RS-values of 0.7, 0.77, 0.84, and 0.91 for HYPER Iterative and Str-values of 2, 3, and 4 for uAI HYPER DPR were selected as the preferred image reconstruction parameters for the clinical study.

### Clinical evaluation

A total of 13 BC patients with 23 IMLN metastatic lymph nodes were included in this evaluation. The BC characteristics of the patients are shown in Table 1. Of these patients, 12 were treated with chemotherapy and 1 with surgical resection as initial treatment.

**Table 1:** Patient characteristics

Age (y/o)	BC characteristic	*n* = 13	
	Median (range)	58 (36–78)	
Histopathology	IDC	10	
	Special Type	apocrine ca.	1
		mucinous ca.	1
	Unknown	1	
Subtype	Luminal A	2	
	Luminal B	2	
	Luminal-HER2	2	
	HER2	3	
	TN	4	
cStage	IIIA/IIIB/IIIC/IV	2/1/8/2	
Histological grade	II/III/unknown	7/5/1	
MIB-1 Index	< 20/≥20/unknown	3/5/5	

**Fig. 5:**
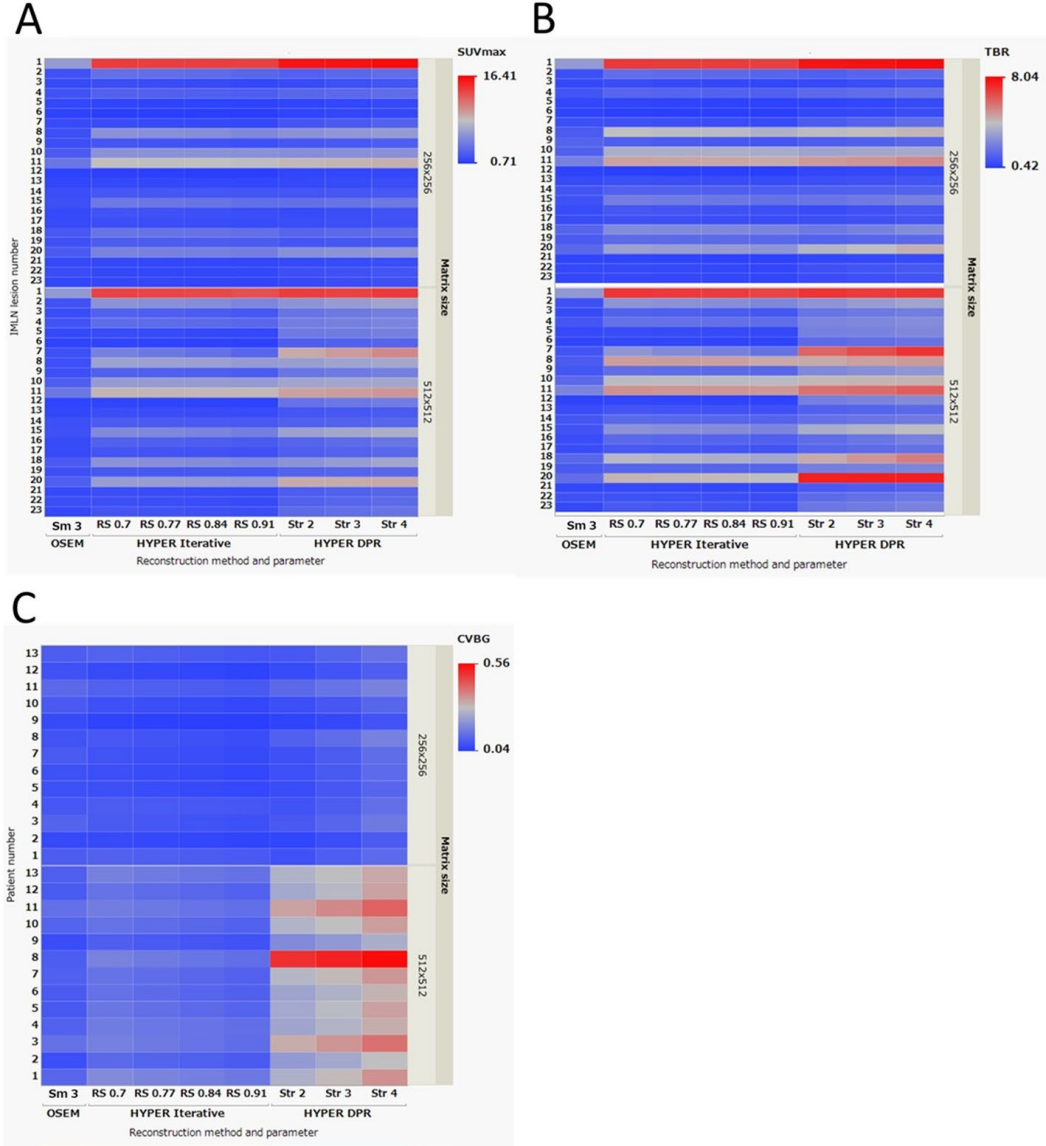
Results of the quantitative evaluation of PET/CT clinical images reconstructed with OSEM, HYPER Iterative, and uAI HYPER DPR, shown as 2D heatmaps. **A** SUV_max_ and **B** tumor-to-background ratio (TBR) of internal mammary lymph node (IMLN) metastases (lesion-based, *n* = 23), and **C** coefficient of variation of the background (CV_BG_) (patient-based, *n* = 13) are shown. The upper and lower rows show the results for the 256 × 256 and 512 × 512 matrix sizes, respectively. The x-axis represents the reconstruction method and parameter (OSEM: Smooth 3; HYPER Iterative: RS values; uAI HYPER DPR: Str values), and the y-axis represents the IMLN metastasis number or patient number

SUV_max_, tumor-to-background ratio (TBR), and background coefficient of variation (CV_BG_) were assessed for images reconstructed with both 256 × 256 and 512 × 512 matrix sizes (Fig. 5). For clarity, representative quantitative results are reported here for the 256 × 256 matrix. With a 256 × 256 matrix size, SUV_max_ of IMLN metastases reconstructed using OSEM ranged from 0.94 to 6.37, whereas that for HYPER Iterative ranged from 0.76 to 14.29 and that for uAI HYPER DPR ranged from 1.13 to 16.41, depending on the reconstruction parameters (Fig. 5A). Similarly, TBR of IMLN metastases reconstructed using OSEM ranged from 0.54 to 3.14, whereas that for HYPER Iterative ranged from 0.42 to 7.08 and that for uAI HYPER DPR ranged from 0.64 to 8.04 across reconstruction parameters (Fig. 5B). For background noise, patient-based CV_BG_ for OSEM ranged from 0.06 to 0.12, whereas that for HYPER Iterative ranged from 0.04 to 0.11 and that for uAI HYPER DPR ranged from 0.04 to 0.12 across reconstruction parameters (Fig. 5C).

**Fig. 6:**
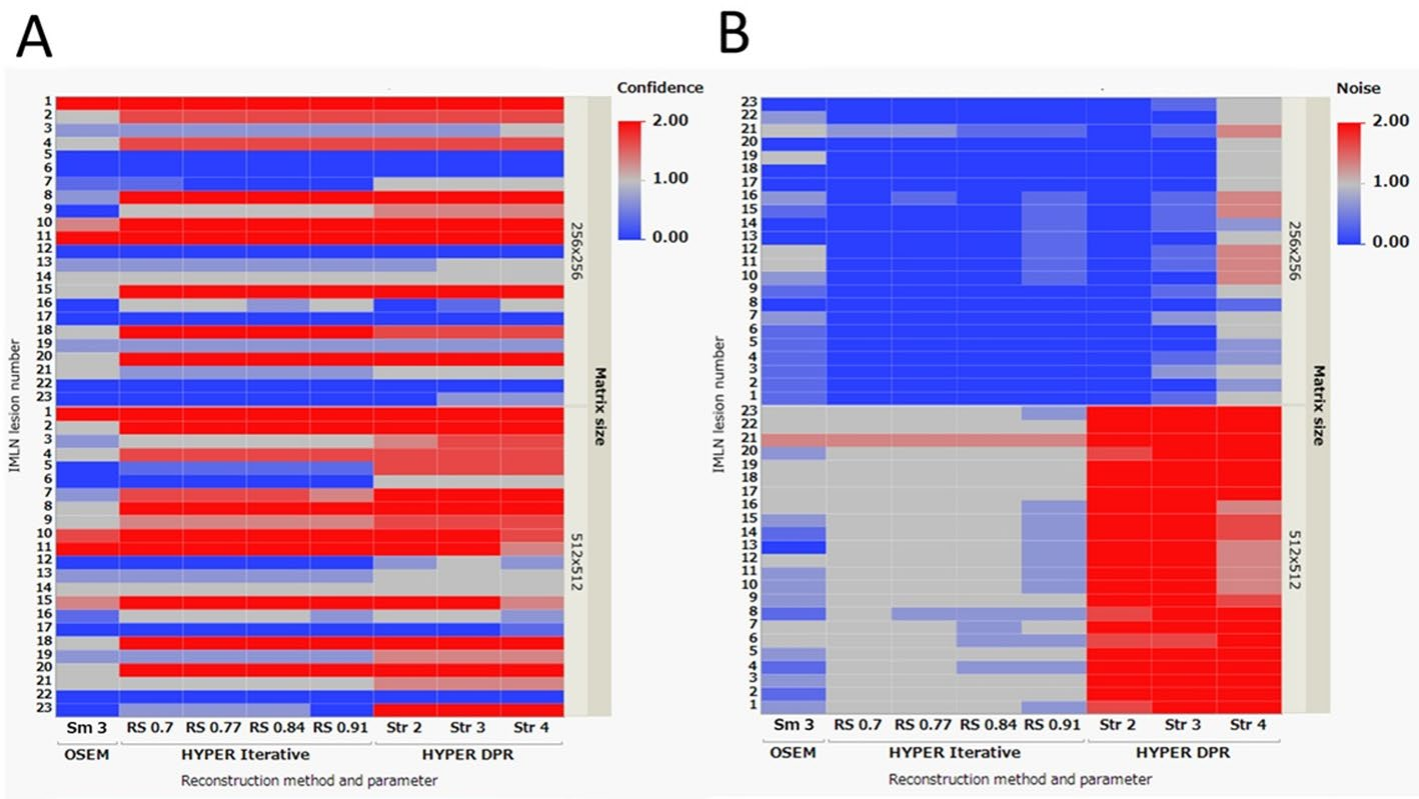
Results of the qualitative evaluation of PET/CT clinical images reconstructed with OSEM, HYPER Iterative, and uAI HYPER DPR, shown as 2D heatmaps. **A** Confidence of internal mammary lymph node (IMLN) metastasis and **B** perceived image noise level are shown (lesion-based, *n* = 23). The upper and lower rows correspond to the 256 × 256 and 512 × 512 matrix sizes, respectively. The x-axis represents the reconstruction method and parameter (OSEM: Smooth 3; HYPER Iterative: RS values; uAI HYPER DPR: Str values), and the y-axis represents the IMLN metastasis number. Color intensity represents the average visual score of three readers based on a discrete three-point scale; a continuous color gradient is used for visualization purposes

In the qualitative evaluation (Fig. 6), higher confidence scores for IMLN metastasis were observed in images reconstructed using HYPER Iterative and uAI HYPER DPR compared with those reconstructed using OSEM. Regarding image noise, uAI HYPER DPR images showed higher noise scores than OSEM and HYPER Iterative across the reconstruction parameters tested. In addition, images reconstructed with a 512 × 512 matrix size showed higher noise scores than those reconstructed with a 256 × 256 matrix size. The Cohen’s kappa values for inter-reader agreement were 0.6733 for confidence of IMLN metastasis and 0.6764 for noise level, indicating substantial agreement [24].

**Fig. 7:**
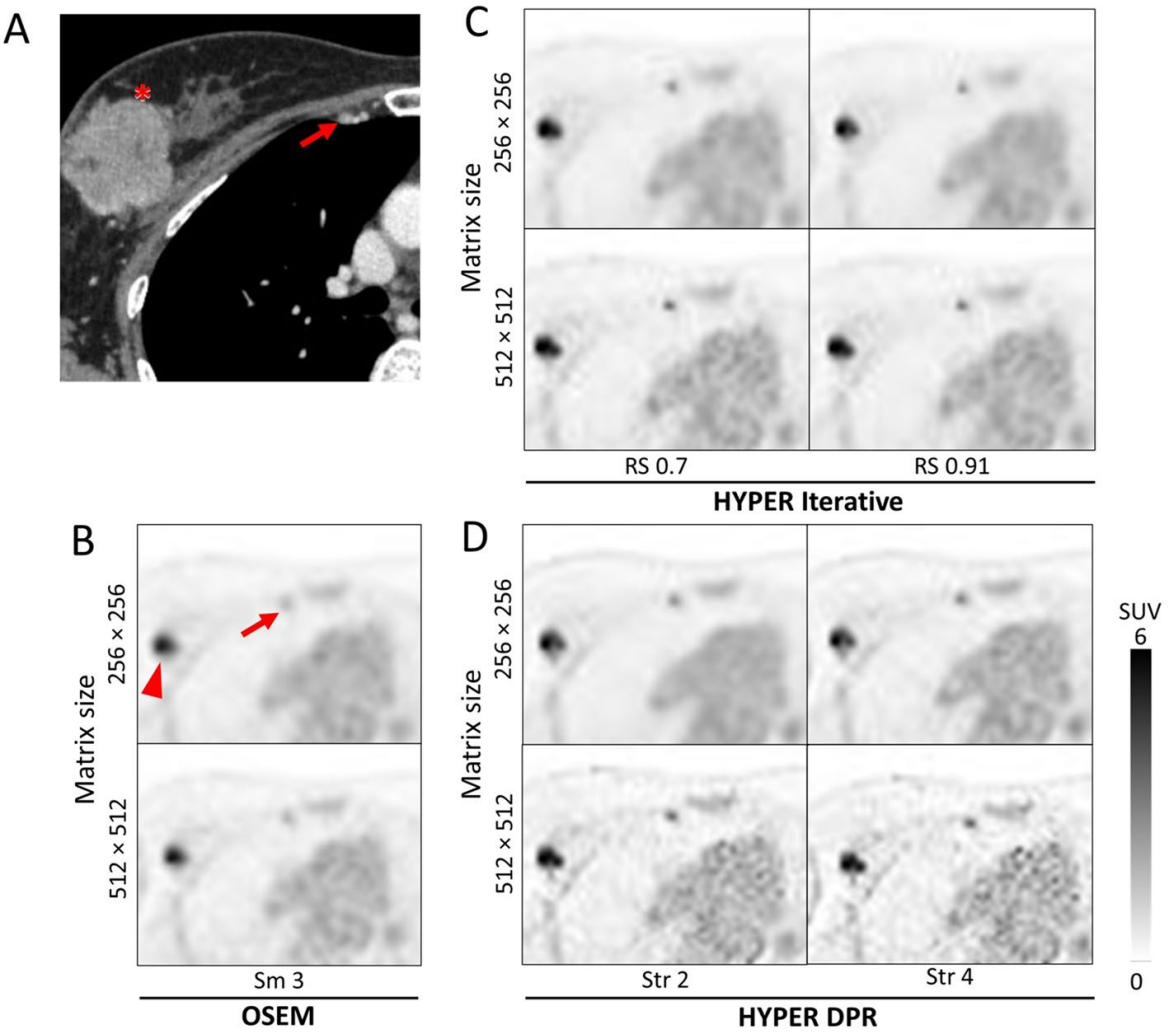
Representative clinical images of a 52 y/o patient with right breast cancer (triple-negative, cT3N2bM1[OSS, LYM]). **A** Contrast-enhanced CT image demonstrating the primary breast cancer (asterisk) and the right internal mammary lymph node (IMLN) metastasis (arrow). **B**–**D** Transverse ^18^F-FDG PET images reconstructed using **B** OSEM (Smooth 3), **C** HYPER Iterative (RS: = 0.7 and 0.91), and **D** uAI HYPER DPR (Str = 2 and 4). The arrowhead in the PET images indicates the right axillary lymph node metastasis. For each reconstruction method, the upper and lower rows correspond to matrix sizes of 256 × 256 and 512 × 512, respectively. Quantitative values (SUV_max_, tumor-to-background ratio [TBR], and background coefficient of variation [CV_BG_]) are shown within each PET image. This case corresponds to IMLN metastasis No. 6 in the lesion-based evaluation and to patient No. 3 in the patient-based evaluation shown in Figs. 5 and 6. Apparent spatial misalignment between the contrast-enhanced CT and PET images is attributable to different scanning positions, with the CT acquired with the arms raised and the PET acquired with the arms lowered. Consequently, the relative position of the primary breast cancer lesion and axillary lymph node metastasis differed depending on the arm position during scanning; however, IMLN metastases fixed to the chest wall were identified in the same position in all images

A representative clinical case illustrating the visual characteristics of each reconstruction method is shown in Fig. 7. In this case, PET images reconstructed using HYPER Iterative and uAI HYPER DPR visualized the IMLN metastasis more clearly than those reconstructed using OSEM. Across all reconstruction methods, images reconstructed with a 512 × 512 matrix size exhibited higher image noise than those with a 256 × 256 matrix size, with this tendency being more pronounced in uAI HYPER DPR across the reconstruction parameters evaluated.

## Discussion

The present study specifically focused on IMLN metastases in BC, which are clinically difficult to identify due to their small lesion size and low FDG uptake, and evaluated advanced image reconstruction methods, including HYPER Iterative and uAI HYPER DPR, on high-resolution digital TOF PET/CT images acquired on a uMI 550 PET/CT system using both phantom and clinical data.

In the phantom evaluation, both HYPER Iterative and uAI HYPER DPR demonstrated higher lesion-to-background contrast than OSEM, while background variability and recovery coefficient varied depending on reconstruction parameters. The reconstruction parameters applied in the clinical analysis were selected based on the phantom evaluation. In the subsequent clinical study, both HYPER Iterative and uAI HYPER DPR showed higher lesion conspicuity than OSEM in detecting smaller IMLN metastases. While smaller RS-values (i.e., β-values) for HYPER Iterative and larger Str-values for uAI HYPER DPR were associated with higher TBR, the noise level increased as a result. However, the effects of these parameters on the confidence of IMLN metastasis detection were minimal. These results were consistent with the previous studies, which have reported the effects of Bayesian penalized likelihood (BPL) and deep learning-based reconstruction methods on PET image noise, contrast recovery, and quantitative stability in PET imaging compared to conventional OSEM reconstruction [17, 20]. In addition, images reconstructed with a 512 × 512 matrix size showed higher background variability (CV_BG_) compared with those reconstructed with a 256 × 256 matrix size, suggesting that the 512 × 512 matrix may not be universally optimal for our clinical use when using our in-house clinical protocol, which has a relatively short acquisition time. The choice of matrix size involves a trade-off between spatial sampling, lesion detectability, and image noise. While increasing matrix size reduces pixel dimensions and may improve visual delineation of small structures, it also decreases counts per voxel, resulting in increased noise and reduced quantitative stability under routine clinical conditions [22, 25]. For reference, the uMI 550 PET/CT system has a transaxial field of view of 70 cm and a detector crystal size of 2.76 mm. With a 512 × 512 matrix, the resulting pixel size is approximately 1.4 mm, which already provides sufficient spatial sampling relative to the intrinsic detector resolution. Further increasing the matrix size would therefore primarily amplify image noise without a meaningful gain in spatial resolution performance. Accordingly, a 512 × 512 matrix was considered a practical upper limit for the present whole-body ^18^F-FDG PET/CT protocol when balancing spatial sampling and noise robustness.

The opposite behavior observed between HYPER Iterative and uAI HYPER DPR reflects fundamental differences in how their control parameters influence the reconstruction process. In HYPER Iterative, the reconstruction strength (RS) parameter directly regulates the strength of the total variation-based regularization during iterative convergence. Lower RS values correspond to weaker regularization, allowing more effective iterations to proceed, which results in higher local contrast and increased SUV_max_ at the expense of greater noise propagation. In contrast, uAI HYPER DPR employs a hybrid reconstruction framework in which convolutional neural networks are embedded within the iterative loop. The Str controls the degree to which the CNN-based denoising and enhancement modules influence the reconstructed image. Higher Str values correspond to stronger CNN intervention, leading to enhanced lesion contrast and increased SUV_max_ while simultaneously suppressing background noise. This Str-dependent behavior is in line with previous reports on HYPER DPR, which demonstrated that increasing the reconstruction strength enhances lesion contrast and SUV-related metrics while maintaining background uniformity through CNN-based denoising, particularly in small-lesion or low-count settings [17].

Although this study was performed using a uMI 550 PET/CT system, the reconstruction principles of HYPER Iterative and uAI HYPER DPR are shared across United Imaging digital PET platforms. Previous studies using other systems, including the uEXPLORER, have reported similar parameter-dependent behavior and trade-offs between contrast enhancement and noise suppression, suggesting that the findings of the present study may be transferable to other United Imaging systems under comparable acquisition conditions [25].

The present study was conducted under a routine clinical acquisition protocol, and the effects of shortened acquisition time or reduced injected activity were not directly evaluated. Nevertheless, the parameter-dependent behavior observed in the phantom study provides a framework for interpreting how these reconstruction methods may respond to changes in count statistics. Both the RS in HYPER Iterative and the Str in uAI HYPER DPR regulate the balance between contrast recovery and noise suppression, which represents a fundamental reconstruction property independent of absolute event counts. Under lower-count conditions, stronger regularization in HYPER Iterative (higher RS) or lower Str values in uAI HYPER DPR would be expected to stabilize image noise at the expense of contrast [26]. However, any modification of the scan protocol would necessitate re-optimization of reconstruction parameters, and the present findings should be regarded as a practical reference for parameter selection rather than as fixed optimal settings.

As with other advanced reconstruction methods, image sharpness and noise remain a trade-off that depends on the clinical task and acquisition conditions [22, 27]. Here, the term “clinical tasks” refers not only to diagnostic purposes, but also includes practical and operational constraints in routine clinical PET imaging, such as time limitations per patient, variability in patient-related factors affecting the count rate (e.g., glucose metabolism or body habitus), and differences in the FDG administered (in-house preparation or supplied radiopharmaceuticals). Despite these sources of variability, our results obtained using the uMI 550 system under the clinical protocol for ^18^F-FDG PET/CT suggested that a certain level of regularization (HYPER iterative method: RS = 0.7–0.84, uAI HYPER DPR: Str = 2–4) provides a reasonable trade-off between lesion contrast and background noise in daily clinical practice. In contrast, when a limited area such as the “internal mammary lymphatic region” is specifically targeted, a large matrix size can be a useful option because it allows clearer delineation of smaller lymph node metastases, despite the increased noise. The IMLN metastases, some of which were difficult to identify using any of the reconstruction parameters, were found to have the following characteristics: (1) very small size, (2) very low FDG accumulation, or (3) both (e.g., numbers 5, 6, 12, and 17 in Figs. 5 and 6). Some of these were only visualized in the uAI HYPER DPR images. It should be noted that this visualization does not necessarily indicate higher true tracer uptake. Rather, these findings appeared as focal, point-like uptakes close to the noise level, and it cannot be conclusively determined whether they represent true annihilation events originating from metastatic lymph nodes or spatially accentuated noise. Conversely, noise seen in normal tissue increases the risk of overdiagnosis and misdiagnosis. Therefore, diagnostic radiologists need to have sufficient knowledge on not only PET image reading but also on BC pathology in order to appropriately leverage advanced PET image reconstruction methods in clinical practice. In particular, focal noise amplification in anatomically complex regions may mimic pathological uptake, leading to false-positive interpretation, especially for small lymph nodes with borderline uptake values. Differentiating such noise-related focal signals from true metastatic involvement requires careful correlation with anatomical structures (e.g., internal mammary vessels) and an understanding of typical metastatic patterns and pathological behavior of BC. In addition, it may be possible to mitigate the impact of high noise and reduce the risk of overdiagnosis using diagnostic support AI [28]. An accurate diagnosis of IMLN metastasis may potentially contribute to treatment decision-making for BC patients, such as applying management as a high-risk group for BC recurrence and setting the irradiation field to include the internal mammary region for postoperative radiotherapy.

In this study, it was not evaluated whether HYPER Iterative or uAI HYPER DPR was more suitable for diagnosing BC metastasis. To our knowledge, this study represents an early clinical evaluation demonstrating the utility of HYPER Iterative and uAI HYPER DPR using clinical BC patient data. Future analysis on a larger patient cohort is likely to clarify the usefulness and the optimal use of advanced PET image reconstruction methods for BC patient care.

In the quantitative analysis of clinical images, the SUV_max_ differences between OSEM and the other two algorithms for IMLN metastasis varied depending on the lymph node. This is consistent with the results of a previous study [29]. The increased SUV_max_ with HYPER Iterative and uAI HYPER DPR was more pronounced for smaller IMLN metastases. While a high-resolution PET/CT system itself can reduce partial volume effects irrespective of the reconstruction algorithm, including OSEM, the additional increase in SUV_max_ compared with OSEM is likely attributable to differences in reconstruction characteristics. In particular, advanced reconstruction methods incorporating penalized likelihood modeling or AI-based components can better preserve or recover focal activity concentration by suppressing background noise and enhancing local contrast, whereas OSEM tends to underestimate uptake in small lesions under similar noise constraints. For small lesions close to the system spatial resolution, conventional reconstruction may underestimate uptake, whereas advanced reconstruction methods can partially recover activity concentration. As a result, this may suggest that SUV_max_ can be more sensitive to parameter tuning when utilizing these algorithms on newer systems for smaller tumors, which may require more thoughtful interpretation of the quantitative PET metrics. Therefore, visual assessment should remain an important evaluation criterion, in addition to quantitative metrics, especially for small metastases. Future research should focus on further understanding these effects and optimizing the reconstruction parameters accordingly.

The present study has several limitations. First, it involved a small number of patients and IMLN metastases. Therefore, it was not possible to statistically indicate the difference between the image reconstruction methods. Nevertheless, it provides basic insights into how HYPER Iterative and uAI HYPER DPR influence image quality and quantitative performance for smaller metastases and the background. In addition, OSEM reconstruction was performed using a routine clinical preset including a 6-mm Gaussian post-smoothing filter, rather than an optimized OSEM setting. While this was intended to reflect a real-world conventional baseline, different post-smoothing parameters may influence image characteristics and quantitative values, and this should be considered when interpreting the results. Second, all diagnoses of IMLN metastases included in this study were based on comprehensive clinical diagnoses, not histopathological diagnoses. However, since IMLN metastases are usually diagnosed by imaging and follow-up, and are not often biopsied or resected clinically, it may be an acceptable approach. In addition, this study did not evaluate the combined effects of reducing both in-plane voxel size and slice thickness. The slice thickness was kept constant to isolate the effect of in-plane voxel size, which may have limited further improvements in axial resolution. Third, this study was conducted at a single institution. Further examinations integrating data from multiple facilities will increase the reliability of the results.

## Conclusions

Our phantom and clinical evaluations have demonstrated that the advanced reconstruction methods—HYPER Iterative and uAI HYPER DPR—achieved higher lesion conspicuity and contrast-related metrics for IMLN metastases compared to OSEM, particularly for smaller metastases. uAI HYPER DPR enabled clearer visualization of smaller metastases, while HYPER Iterative provided improved noise suppression. Achieving appropriate image quality requires careful selection of reconstruction parameters to balance contrast and noise, highlighting the importance of acquisition protocol design and parameter optimization in advanced PET reconstruction techniques. These findings suggest the potential for such advanced reconstruction methods to contribute towards improved clinical assessment of IMLN metastases in BC imaging.

## Supplementary Information

Below is the link to the electronic supplementary material.

Supplementary Material 1

## Data Availability

The datasets used and/or analyzed during the current study are available from the corresponding author upon reasonable request.
