# Supplementary material for: Advanced image reconstruction algorithms for high-resolution digital time-of-flight PET/CT enhance visualization of sub-clinical internal mammary lymph node metastases in breast cancer: a phantom and a clinical, retrospective cohort study
Source: EJNMMI Phys. 2026 Feb 22;13:22. doi: 10.1186/s40658-026-00846-8 (PMC12963589; doi:10.1186/s40658-026-00846-8)
Supplement: Supplementary file 1 — Supplementary Material 1 [file 40658_2026_846_MOESM1_ESM.docx]

**Supplementary Table S1. Lesion-based quantitative results of IMLN metastases on clinical PET/CT images**

| IMLN No. | Matrix size | Reconstruction | Parameter | SUV_max_ | TBR |
| --- | --- | --- | --- | --- | --- |
| 1 | 256x256 | OSEM | Smooth 3 | 6.3725319 | 3.1416945 |
| 1 | 256x256 | HYPER Iterative | RS 0.7 | 14.292665 | 7.0823507 |
| 1 | 256x256 | HYPER Iterative | RS 0.77 | 14.187817 | 7.0232333 |
| 1 | 256x256 | HYPER Iterative | RS 0.84 | 14.187817 | 7.0178253 |
| 1 | 256x256 | HYPER Iterative | RS 0.91 | 14.008643 | 6.9281346 |
| 1 | 256x256 | HYPER DPR | Str 2 | 15.98646 | 7.8570928 |
| 1 | 256x256 | HYPER DPR | Str 3 | 16.051435 | 7.8917838 |
| 1 | 256x256 | HYPER DPR | Str 4 | 16.414709 | 8.0415256 |
| 1 | 512x512 | OSEM | Smooth 3 | 6.1633301 | 3.0741987 |
| 1 | 512x512 | HYPER Iterative | RS 0.7 | 13.978124 | 7.1335468 |
| 1 | 512x512 | HYPER Iterative | RS 0.77 | 13.849649 | 7.0582408 |
| 1 | 512x512 | HYPER Iterative | RS 0.84 | 13.740372 | 6.9924192 |
| 1 | 512x512 | HYPER Iterative | RS 0.91 | 13.40368 | 6.8106711 |
| 1 | 512x512 | HYPER DPR | Str 2 | 14.215405 | 7.1522671 |
| 1 | 512x512 | HYPER DPR | Str 3 | 13.947853 | 7.0206751 |
| 1 | 512x512 | HYPER DPR | Str 4 | 14.38469 | 7.1017282 |
| 2 | 256x256 | OSEM | Smooth 3 | 1.8390067 | 0.9066407 |
| 2 | 256x256 | HYPER Iterative | RS 0.7 | 3.7095165 | 1.8381525 |
| 2 | 256x256 | HYPER Iterative | RS 0.77 | 3.5987628 | 1.7814545 |
| 2 | 256x256 | HYPER Iterative | RS 0.84 | 3.4870243 | 1.7248128 |
| 2 | 256x256 | HYPER Iterative | RS 0.91 | 3.1685452 | 1.5670403 |
| 2 | 256x256 | HYPER DPR | Str 2 | 2.8431752 | 1.3973758 |
| 2 | 256x256 | HYPER DPR | Str 3 | 3.1941419 | 1.5704189 |
| 2 | 256x256 | HYPER DPR | Str 4 | 3.5524921 | 1.7403572 |
| 2 | 512x512 | OSEM | Smooth 3 | 1.8941374 | 0.9447741 |
| 2 | 512x512 | HYPER Iterative | RS 0.7 | 5.8099022 | 2.9650052 |
| 2 | 512x512 | HYPER Iterative | RS 0.77 | 5.645494 | 2.877131 |
| 2 | 512x512 | HYPER Iterative | RS 0.84 | 5.482563 | 2.790054 |
| 2 | 512x512 | HYPER Iterative | RS 0.91 | 5.0498848 | 2.5659449 |
| 2 | 512x512 | HYPER DPR | Str 2 | 5.8536501 | 2.945176 |
| 2 | 512x512 | HYPER DPR | Str 3 | 6.3576479 | 3.2001327 |
| 2 | 512x512 | HYPER DPR | Str 4 | 6.9406242 | 3.4265894 |
| 3 | 256x256 | OSEM | Smooth 3 | 1.4629357 | 0.7212356 |
| 3 | 256x256 | HYPER Iterative | RS 0.7 | 1.6657383 | 0.8254124 |
| 3 | 256x256 | HYPER Iterative | RS 0.77 | 1.5303724 | 0.7575628 |
| 3 | 256x256 | HYPER Iterative | RS 0.84 | 1.4166651 | 0.7007356 |
| 3 | 256x256 | HYPER Iterative | RS 0.91 | 1.3068957 | 0.6463402 |
| 3 | 256x256 | HYPER DPR | Str 2 | 1.496408 | 0.7354609 |
| 3 | 256x256 | HYPER DPR | Str 3 | 1.7745231 | 0.8724549 |
| 3 | 256x256 | HYPER DPR | Str 4 | 2.0767584 | 1.0173988 |
| 3 | 512x512 | OSEM | Smooth 3 | 1.5751662 | 0.7856749 |
| 3 | 512x512 | HYPER Iterative | RS 0.7 | 2.8653259 | 1.4622804 |
| 3 | 512x512 | HYPER Iterative | RS 0.77 | 2.6723678 | 1.3619273 |
| 3 | 512x512 | HYPER Iterative | RS 0.84 | 2.493685 | 1.2690261 |
| 3 | 512x512 | HYPER Iterative | RS 0.91 | 2.2544565 | 1.1455333 |
| 3 | 512x512 | HYPER DPR | Str 2 | 3.9905016 | 2.0077609 |
| 3 | 512x512 | HYPER DPR | Str 3 | 4.2891288 | 2.1589401 |
| 3 | 512x512 | HYPER DPR | Str 4 | 4.6326408 | 2.2871369 |
| 4 | 256x256 | OSEM | Smooth 3 | 1.9072624 | 1.0634117 |
| 4 | 256x256 | HYPER Iterative | RS 0.7 | 2.8414228 | 1.5685034 |
| 4 | 256x256 | HYPER Iterative | RS 0.77 | 2.77667 | 1.5305838 |
| 4 | 256x256 | HYPER Iterative | RS 0.84 | 2.7146342 | 1.4963878 |
| 4 | 256x256 | HYPER Iterative | RS 0.91 | 2.5892041 | 1.4221834 |
| 4 | 256x256 | HYPER DPR | Str 2 | 3.1429064 | 1.7161035 |
| 4 | 256x256 | HYPER DPR | Str 3 | 3.2912817 | 1.8085985 |
| 4 | 256x256 | HYPER DPR | Str 4 | 3.5129261 | 1.9519775 |
| 4 | 512x512 | OSEM | Smooth 3 | 1.9837885 | 1.1055756 |
| 4 | 512x512 | HYPER Iterative | RS 0.7 | 3.4936137 | 1.9294599 |
| 4 | 512x512 | HYPER Iterative | RS 0.77 | 3.4272599 | 1.8948319 |
| 4 | 512x512 | HYPER Iterative | RS 0.84 | 3.3625088 | 1.8607605 |
| 4 | 512x512 | HYPER Iterative | RS 0.91 | 3.2221048 | 1.7822831 |
| 4 | 512x512 | HYPER DPR | Str 2 | 4.5205832 | 2.4929081 |
| 4 | 512x512 | HYPER DPR | Str 3 | 4.6739039 | 2.5663721 |
| 4 | 512x512 | HYPER DPR | Str 4 | 4.9534183 | 2.7192089 |
| 5 | 256x256 | OSEM | Smooth 3 | 1.1881902 | 0.6624864 |
| 5 | 256x256 | HYPER Iterative | RS 0.7 | 1.0686467 | 0.5899073 |
| 5 | 256x256 | HYPER Iterative | RS 0.77 | 1.0260819 | 0.5656071 |
| 5 | 256x256 | HYPER Iterative | RS 0.84 | 0.9907622 | 0.5461379 |
| 5 | 256x256 | HYPER Iterative | RS 0.91 | 0.9604235 | 0.527536 |
| 5 | 256x256 | HYPER DPR | Str 2 | 1.0526438 | 0.5747692 |
| 5 | 256x256 | HYPER DPR | Str 3 | 1.18106 | 0.6490065 |
| 5 | 256x256 | HYPER DPR | Str 4 | 1.3879485 | 0.7712216 |
| 5 | 512x512 | OSEM | Smooth 3 | 1.3394308 | 0.7464718 |
| 5 | 512x512 | HYPER Iterative | RS 0.7 | 1.5121386 | 0.8351269 |
| 5 | 512x512 | HYPER Iterative | RS 0.77 | 1.4190687 | 0.7845616 |
| 5 | 512x512 | HYPER Iterative | RS 0.84 | 1.3410619 | 0.742123 |
| 5 | 512x512 | HYPER Iterative | RS 0.91 | 1.2635237 | 0.6989087 |
| 5 | 512x512 | HYPER DPR | Str 2 | 4.3032327 | 2.3730486 |
| 5 | 512x512 | HYPER DPR | Str 3 | 4.4437017 | 2.4399714 |
| 5 | 512x512 | HYPER DPR | Str 4 | 4.6974812 | 2.5787107 |
| 6 | 256x256 | OSEM | Smooth 3 | 0.9371215 | 0.5634214 |
| 6 | 256x256 | HYPER Iterative | RS 0.7 | 0.7727143 | 0.4721183 |
| 6 | 256x256 | HYPER Iterative | RS 0.77 | 0.7438184 | 0.452678 |
| 6 | 256x256 | HYPER Iterative | RS 0.84 | 0.7233921 | 0.4386459 |
| 6 | 256x256 | HYPER Iterative | RS 0.91 | 0.7059549 | 0.4266883 |
| 6 | 256x256 | HYPER DPR | Str 2 | 1.0925611 | 0.6545096 |
| 6 | 256x256 | HYPER DPR | Str 3 | 1.1807432 | 0.7197945 |
| 6 | 256x256 | HYPER DPR | Str 4 | 1.3307027 | 0.8249015 |
| 6 | 512x512 | OSEM | Smooth 3 | 1.0038809 | 0.6133802 |
| 6 | 512x512 | HYPER Iterative | RS 0.7 | 1.0470412 | 0.6604439 |
| 6 | 512x512 | HYPER Iterative | RS 0.77 | 0.9921039 | 0.6236801 |
| 6 | 512x512 | HYPER Iterative | RS 0.84 | 0.945162 | 0.5921444 |
| 6 | 512x512 | HYPER Iterative | RS 0.91 | 0.8987559 | 0.5608463 |
| 6 | 512x512 | HYPER DPR | Str 2 | 2.6718676 | 1.7343499 |
| 6 | 512x512 | HYPER DPR | Str 3 | 2.8233216 | 1.8496073 |
| 6 | 512x512 | HYPER DPR | Str 4 | 3.03456 | 1.993536 |
| 7 | 256x256 | OSEM | Smooth 3 | 1.5135434 | 0.909981 |
| 7 | 256x256 | HYPER Iterative | RS 0.7 | 1.5713352 | 0.9600652 |
| 7 | 256x256 | HYPER Iterative | RS 0.77 | 1.4592392 | 0.8880736 |
| 7 | 256x256 | HYPER Iterative | RS 0.84 | 1.3645805 | 0.8274456 |
| 7 | 256x256 | HYPER Iterative | RS 0.91 | 1.2818787 | 0.7747842 |
| 7 | 256x256 | HYPER DPR | Str 2 | 2.1223488 | 1.2714142 |
| 7 | 256x256 | HYPER DPR | Str 3 | 2.4362171 | 1.4851457 |
| 7 | 256x256 | HYPER DPR | Str 4 | 2.9055252 | 1.8011327 |
| 7 | 512x512 | OSEM | Smooth 3 | 1.7377353 | 1.0617718 |
| 7 | 512x512 | HYPER Iterative | RS 0.7 | 4.7190585 | 2.9766485 |
| 7 | 512x512 | HYPER Iterative | RS 0.77 | 4.2182112 | 2.6517528 |
| 7 | 512x512 | HYPER Iterative | RS 0.84 | 3.7710905 | 2.3625898 |
| 7 | 512x512 | HYPER Iterative | RS 0.91 | 3.0680463 | 1.9145379 |
| 7 | 512x512 | HYPER DPR | Str 2 | 9.5186834 | 6.1787223 |
| 7 | 512x512 | HYPER DPR | Str 3 | 10.1798 | 6.6689648 |
| 7 | 512x512 | HYPER DPR | Str 4 | 10.956999 | 7.1981348 |
| 8 | 256x256 | OSEM | Smooth 3 | 1.7604094 | 1.2833758 |
| 8 | 256x256 | HYPER Iterative | RS 0.7 | 5.7757311 | 4.2212089 |
| 8 | 256x256 | HYPER Iterative | RS 0.77 | 5.6495214 | 4.1375223 |
| 8 | 256x256 | HYPER Iterative | RS 0.84 | 5.5211358 | 4.0514063 |
| 8 | 256x256 | HYPER Iterative | RS 0.91 | 5.1561065 | 3.7940172 |
| 8 | 256x256 | HYPER DPR | Str 2 | 5.7719231 | 4.0982657 |
| 8 | 256x256 | HYPER DPR | Str 3 | 6.0809193 | 4.3027783 |
| 8 | 256x256 | HYPER DPR | Str 4 | 6.3094025 | 4.448173 |
| 8 | 512x512 | OSEM | Smooth 3 | 1.7272248 | 1.2876472 |
| 8 | 512x512 | HYPER Iterative | RS 0.7 | 6.7047186 | 4.9687478 |
| 8 | 512x512 | HYPER Iterative | RS 0.77 | 6.659945 | 4.9427881 |
| 8 | 512x512 | HYPER Iterative | RS 0.84 | 6.6069617 | 4.9100085 |
| 8 | 512x512 | HYPER Iterative | RS 0.91 | 6.3604212 | 4.7375106 |
| 8 | 512x512 | HYPER DPR | Str 2 | 6.5171213 | 4.6692184 |
| 8 | 512x512 | HYPER DPR | Str 3 | 6.8451982 | 4.9026042 |
| 8 | 512x512 | HYPER DPR | Str 4 | 7.0690928 | 5.0417375 |
| 9 | 256x256 | OSEM | Smooth 3 | 1.7049366 | 1.5355442 |
| 9 | 256x256 | HYPER Iterative | RS 0.7 | 2.1324391 | 1.3568353 |
| 9 | 256x256 | HYPER Iterative | RS 0.77 | 2.0059431 | 1.2784283 |
| 9 | 256x256 | HYPER Iterative | RS 0.84 | 1.8861302 | 1.2039333 |
| 9 | 256x256 | HYPER Iterative | RS 0.91 | 1.7345419 | 1.1092646 |
| 9 | 256x256 | HYPER DPR | Str 2 | 2.1554034 | 1.3319138 |
| 9 | 256x256 | HYPER DPR | Str 3 | 2.3532131 | 1.4636192 |
| 9 | 256x256 | HYPER DPR | Str 4 | 2.5783544 | 1.6075434 |
| 9 | 512x512 | OSEM | Smooth 3 | 1.7533947 | 1.091961 |
| 9 | 512x512 | HYPER Iterative | RS 0.7 | 2.8201718 | 1.8136078 |
| 9 | 512x512 | HYPER Iterative | RS 0.77 | 2.7289855 | 1.7537841 |
| 9 | 512x512 | HYPER Iterative | RS 0.84 | 2.6370404 | 1.6937207 |
| 9 | 512x512 | HYPER Iterative | RS 0.91 | 2.4942458 | 1.6020141 |
| 9 | 512x512 | HYPER DPR | Str 2 | 4.1160359 | 2.6175565 |
| 9 | 512x512 | HYPER DPR | Str 3 | 4.3268695 | 2.7723676 |
| 9 | 512x512 | HYPER DPR | Str 4 | 4.6476068 | 2.9829731 |
| 10 | 256x256 | OSEM | Smooth 3 | 2.5000463 | 1.4019319 |
| 10 | 256x256 | HYPER Iterative | RS 0.7 | 5.8042722 | 3.693161 |
| 10 | 256x256 | HYPER Iterative | RS 0.77 | 5.8081245 | 3.701636 |
| 10 | 256x256 | HYPER Iterative | RS 0.84 | 5.8077927 | 3.7071646 |
| 10 | 256x256 | HYPER Iterative | RS 0.91 | 5.6793823 | 3.632047 |
| 10 | 256x256 | HYPER DPR | Str 2 | 5.5126758 | 3.4065128 |
| 10 | 256x256 | HYPER DPR | Str 3 | 5.622663 | 3.4971068 |
| 10 | 256x256 | HYPER DPR | Str 4 | 5.7212362 | 3.5670564 |
| 10 | 512x512 | OSEM | Smooth 3 | 2.6562991 | 1.6542624 |
| 10 | 512x512 | HYPER Iterative | RS 0.7 | 6.3578987 | 4.088664 |
| 10 | 512x512 | HYPER Iterative | RS 0.77 | 6.3680344 | 4.092421 |
| 10 | 512x512 | HYPER Iterative | RS 0.84 | 6.3714938 | 4.092289 |
| 10 | 512x512 | HYPER Iterative | RS 0.91 | 6.3059006 | 4.0501789 |
| 10 | 512x512 | HYPER DPR | Str 2 | 6.9257951 | 4.4043979 |
| 10 | 512x512 | HYPER DPR | Str 3 | 6.9790907 | 4.471733 |
| 10 | 512x512 | HYPER DPR | Str 4 | 7.0286798 | 4.5112169 |
| 11 | 256x256 | OSEM | Smooth 3 | 4.1239052 | 2.4005956 |
| 11 | 256x256 | HYPER Iterative | RS 0.7 | 8.6625557 | 4.8646975 |
| 11 | 256x256 | HYPER Iterative | RS 0.77 | 8.6150627 | 4.8181857 |
| 11 | 256x256 | HYPER Iterative | RS 0.84 | 8.5687923 | 4.7770846 |
| 11 | 256x256 | HYPER Iterative | RS 0.91 | 8.4070196 | 4.6754321 |
| 11 | 256x256 | HYPER DPR | Str 2 | 8.904233 | 5.0282675 |
| 11 | 256x256 | HYPER DPR | Str 3 | 9.0358143 | 5.1569558 |
| 11 | 256x256 | HYPER DPR | Str 4 | 9.2614403 | 5.3809883 |
| 11 | 512x512 | OSEM | Smooth 3 | 4.2150383 | 2.4825799 |
| 11 | 512x512 | HYPER Iterative | RS 0.7 | 9.0473003 | 5.2162997 |
| 11 | 512x512 | HYPER Iterative | RS 0.77 | 8.9970751 | 5.1615956 |
| 11 | 512x512 | HYPER Iterative | RS 0.84 | 8.9501228 | 5.1128948 |
| 11 | 512x512 | HYPER Iterative | RS 0.91 | 8.9186563 | 5.0736664 |
| 11 | 512x512 | HYPER DPR | Str 2 | 9.8825464 | 5.8684138 |
| 11 | 512x512 | HYPER DPR | Str 3 | 10.038837 | 6.0199359 |
| 11 | 512x512 | HYPER DPR | Str 4 | 10.345819 | 6.2991962 |
| 12 | 256x256 | OSEM | Smooth 3 | 0.9361041 | 0.5449221 |
| 12 | 256x256 | HYPER Iterative | RS 0.7 | 0.8412048 | 0.4724018 |
| 12 | 256x256 | HYPER Iterative | RS 0.77 | 0.810671 | 0.4533877 |
| 12 | 256x256 | HYPER Iterative | RS 0.84 | 0.7838159 | 0.4369758 |
| 12 | 256x256 | HYPER Iterative | RS 0.91 | 0.7596883 | 0.4224887 |
| 12 | 256x256 | HYPER DPR | Str 2 | 1.1276246 | 0.6367756 |
| 12 | 256x256 | HYPER DPR | Str 3 | 1.2102766 | 0.6907339 |
| 12 | 256x256 | HYPER DPR | Str 4 | 1.3073158 | 0.7595634 |
| 12 | 512x512 | OSEM | Smooth 3 | 0.9823717 | 0.5785989 |
| 12 | 512x512 | HYPER Iterative | RS 0.7 | 1.0826535 | 0.6242133 |
| 12 | 512x512 | HYPER Iterative | RS 0.77 | 1.0246555 | 0.5878418 |
| 12 | 512x512 | HYPER Iterative | RS 0.84 | 0.9749576 | 0.5569595 |
| 12 | 512x512 | HYPER Iterative | RS 0.91 | 0.9292325 | 0.528624 |
| 12 | 512x512 | HYPER DPR | Str 2 | 3.8963492 | 2.3137143 |
| 12 | 512x512 | HYPER DPR | Str 3 | 4.1578836 | 2.4933358 |
| 12 | 512x512 | HYPER DPR | Str 4 | 4.4507113 | 2.7098778 |
| 13 | 256x256 | OSEM | Smooth 3 | 1.2035079 | 0.7817446 |
| 13 | 256x256 | HYPER Iterative | RS 0.7 | 1.3750412 | 0.8968311 |
| 13 | 256x256 | HYPER Iterative | RS 0.77 | 1.3277049 | 0.8626235 |
| 13 | 256x256 | HYPER Iterative | RS 0.84 | 1.2823604 | 0.8304672 |
| 13 | 256x256 | HYPER Iterative | RS 0.91 | 1.2334642 | 0.7968496 |
| 13 | 256x256 | HYPER DPR | Str 2 | 1.3910367 | 0.9024156 |
| 13 | 256x256 | HYPER DPR | Str 3 | 1.4741937 | 0.9702033 |
| 13 | 256x256 | HYPER DPR | Str 4 | 1.5988929 | 1.0660366 |
| 13 | 512x512 | OSEM | Smooth 3 | 1.2522372 | 0.8227236 |
| 13 | 512x512 | HYPER Iterative | RS 0.7 | 1.6237017 | 1.0801009 |
| 13 | 512x512 | HYPER Iterative | RS 0.77 | 1.5703621 | 1.0406519 |
| 13 | 512x512 | HYPER Iterative | RS 0.84 | 1.5215666 | 1.0047781 |
| 13 | 512x512 | HYPER Iterative | RS 0.91 | 1.467046 | 0.9656516 |
| 13 | 512x512 | HYPER DPR | Str 2 | 2.1029842 | 1.450927 |
| 13 | 512x512 | HYPER DPR | Str 3 | 2.2060378 | 1.5336082 |
| 13 | 512x512 | HYPER DPR | Str 4 | 2.4361808 | 1.6926302 |
| 14 | 256x256 | OSEM | Smooth 3 | 1.6373692 | 1.0635613 |
| 14 | 256x256 | HYPER Iterative | RS 0.7 | 2.1545658 | 1.4052536 |
| 14 | 256x256 | HYPER Iterative | RS 0.77 | 2.1368585 | 1.3883388 |
| 14 | 256x256 | HYPER Iterative | RS 0.84 | 2.1173549 | 1.3712166 |
| 14 | 256x256 | HYPER Iterative | RS 0.91 | 2.077172 | 1.3419066 |
| 14 | 256x256 | HYPER DPR | Str 2 | 2.1358736 | 1.385618 |
| 14 | 256x256 | HYPER DPR | Str 3 | 2.1975639 | 1.4462711 |
| 14 | 256x256 | HYPER DPR | Str 4 | 2.2462621 | 1.4976598 |
| 14 | 512x512 | OSEM | Smooth 3 | 1.693334 | 1.1125255 |
| 14 | 512x512 | HYPER Iterative | RS 0.7 | 2.4871681 | 1.6544865 |
| 14 | 512x512 | HYPER Iterative | RS 0.77 | 2.4282691 | 1.6091721 |
| 14 | 512x512 | HYPER Iterative | RS 0.84 | 2.3746843 | 1.5681408 |
| 14 | 512x512 | HYPER Iterative | RS 0.91 | 2.3146346 | 1.5235586 |
| 14 | 512x512 | HYPER DPR | Str 2 | 2.7454708 | 1.8942024 |
| 14 | 512x512 | HYPER DPR | Str 3 | 2.9236443 | 2.0324788 |
| 14 | 512x512 | HYPER DPR | Str 4 | 3.1315088 | 2.175736 |
| 15 | 256x256 | OSEM | Smooth 3 | 1.7668809 | 0.9739081 |
| 15 | 256x256 | HYPER Iterative | RS 0.7 | 4.253675 | 2.2853261 |
| 15 | 256x256 | HYPER Iterative | RS 0.77 | 4.1269784 | 2.210176 |
| 15 | 256x256 | HYPER Iterative | RS 0.84 | 3.9997902 | 2.1367438 |
| 15 | 256x256 | HYPER Iterative | RS 0.91 | 3.6034942 | 1.9209298 |
| 15 | 256x256 | HYPER DPR | Str 2 | 3.6923785 | 2.0248768 |
| 15 | 256x256 | HYPER DPR | Str 3 | 3.939388 | 2.1808283 |
| 15 | 256x256 | HYPER DPR | Str 4 | 4.2433624 | 2.3953374 |
| 15 | 512x512 | OSEM | Smooth 3 | 1.8611668 | 1.0322633 |
| 15 | 512x512 | HYPER Iterative | RS 0.7 | 5.3193035 | 2.8948199 |
| 15 | 512x512 | HYPER Iterative | RS 0.77 | 5.1508651 | 2.7976214 |
| 15 | 512x512 | HYPER Iterative | RS 0.84 | 4.9883204 | 2.7049275 |
| 15 | 512x512 | HYPER Iterative | RS 0.91 | 4.6067562 | 2.4917988 |
| 15 | 512x512 | HYPER DPR | Str 2 | 6.6579685 | 3.6601016 |
| 15 | 512x512 | HYPER DPR | Str 3 | 7.0174346 | 3.864617 |
| 15 | 512x512 | HYPER DPR | Str 4 | 7.526186 | 4.1851163 |
| 16 | 256x256 | OSEM | Smooth 3 | 1.397868 | 0.8046389 |
| 16 | 256x256 | HYPER Iterative | RS 0.7 | 1.9582794 | 1.1284951 |
| 16 | 256x256 | HYPER Iterative | RS 0.77 | 1.8415673 | 1.0545639 |
| 16 | 256x256 | HYPER Iterative | RS 0.84 | 1.7310017 | 0.9852048 |
| 16 | 256x256 | HYPER Iterative | RS 0.91 | 1.5911391 | 0.8997394 |
| 16 | 256x256 | HYPER DPR | Str 2 | 1.4321328 | 0.803657 |
| 16 | 256x256 | HYPER DPR | Str 3 | 1.6513733 | 0.9477565 |
| 16 | 256x256 | HYPER DPR | Str 4 | 1.9508634 | 1.1401018 |
| 16 | 512x512 | OSEM | Smooth 3 | 1.4276675 | 0.824492 |
| 16 | 512x512 | HYPER Iterative | RS 0.7 | 2.849494 | 1.6786292 |
| 16 | 512x512 | HYPER Iterative | RS 0.77 | 2.7053092 | 1.5886403 |
| 16 | 512x512 | HYPER Iterative | RS 0.84 | 2.5700264 | 1.5042952 |
| 16 | 512x512 | HYPER Iterative | RS 0.91 | 2.3890543 | 1.3920934 |
| 16 | 512x512 | HYPER DPR | Str 2 | 3.0308697 | 1.7816967 |
| 16 | 512x512 | HYPER DPR | Str 3 | 3.4471343 | 2.0366059 |
| 16 | 512x512 | HYPER DPR | Str 4 | 4.0324416 | 2.3821832 |
| 17 | 256x256 | OSEM | Smooth 3 | 1.4623433 | 0.8417521 |
| 17 | 256x256 | HYPER Iterative | RS 0.7 | 1.6491864 | 0.9503745 |
| 17 | 256x256 | HYPER Iterative | RS 0.77 | 1.5940077 | 0.9128002 |
| 17 | 256x256 | HYPER Iterative | RS 0.84 | 1.541481 | 0.8773385 |
| 17 | 256x256 | HYPER Iterative | RS 0.91 | 1.4872664 | 0.8410027 |
| 17 | 256x256 | HYPER DPR | Str 2 | 1.6121012 | 0.9046482 |
| 17 | 256x256 | HYPER DPR | Str 3 | 1.7123775 | 0.9827681 |
| 17 | 256x256 | HYPER DPR | Str 4 | 1.8547401 | 1.0839266 |
| 17 | 512x512 | OSEM | Smooth 3 | 1.4639688 | 0.8454563 |
| 17 | 512x512 | HYPER Iterative | RS 0.7 | 2.0379784 | 1.2005676 |
| 17 | 512x512 | HYPER Iterative | RS 0.77 | 1.9575859 | 1.1495543 |
| 17 | 512x512 | HYPER Iterative | RS 0.84 | 1.8846393 | 1.1031224 |
| 17 | 512x512 | HYPER Iterative | RS 0.91 | 1.7973597 | 1.0473151 |
| 17 | 512x512 | HYPER DPR | Str 2 | 2.6819062 | 1.5765585 |
| 17 | 512x512 | HYPER DPR | Str 3 | 2.9210167 | 1.7257697 |
| 17 | 512x512 | HYPER DPR | Str 4 | 3.2353308 | 1.9112864 |
| 18 | 256x256 | OSEM | Smooth 3 | 2.25823 | 1.5581889 |
| 18 | 256x256 | HYPER Iterative | RS 0.7 | 3.9722018 | 2.6882307 |
| 18 | 256x256 | HYPER Iterative | RS 0.77 | 3.920424 | 2.6305727 |
| 18 | 256x256 | HYPER Iterative | RS 0.84 | 3.8686459 | 2.5759368 |
| 18 | 256x256 | HYPER Iterative | RS 0.91 | 3.710958 | 2.4509512 |
| 18 | 256x256 | HYPER DPR | Str 2 | 3.1019773 | 2.1434401 |
| 18 | 256x256 | HYPER DPR | Str 3 | 3.3432162 | 2.3886863 |
| 18 | 256x256 | HYPER DPR | Str 4 | 3.6503541 | 2.7114228 |
| 18 | 512x512 | OSEM | Smooth 3 | 2.3676701 | 1.6197252 |
| 18 | 512x512 | HYPER Iterative | RS 0.7 | 5.768548 | 4.0056003 |
| 18 | 512x512 | HYPER Iterative | RS 0.77 | 5.6073298 | 3.867733 |
| 18 | 512x512 | HYPER Iterative | RS 0.84 | 5.4525843 | 3.7380538 |
| 18 | 512x512 | HYPER Iterative | RS 0.91 | 5.1519184 | 3.5050331 |
| 18 | 512x512 | HYPER DPR | Str 2 | 5.8632784 | 4.6986337 |
| 18 | 512x512 | HYPER DPR | Str 3 | 6.3369303 | 5.0917876 |
| 18 | 512x512 | HYPER DPR | Str 4 | 6.9781785 | 5.622442 |
| 19 | 256x256 | OSEM | Smooth 3 | 1.8198816 | 1.2557265 |
| 19 | 256x256 | HYPER Iterative | RS 0.7 | 2.5518353 | 1.7269822 |
| 19 | 256x256 | HYPER Iterative | RS 0.77 | 2.5406559 | 1.7047595 |
| 19 | 256x256 | HYPER Iterative | RS 0.84 | 2.5277114 | 1.6830759 |
| 19 | 256x256 | HYPER Iterative | RS 0.91 | 2.4659309 | 1.6286566 |
| 19 | 256x256 | HYPER DPR | Str 2 | 2.1293733 | 1.4713789 |
| 19 | 256x256 | HYPER DPR | Str 3 | 2.1917424 | 1.5659726 |
| 19 | 256x256 | HYPER DPR | Str 4 | 2.2482276 | 1.6699463 |
| 19 | 512x512 | OSEM | Smooth 3 | 1.8198816 | 1.2449826 |
| 19 | 512x512 | HYPER Iterative | RS 0.7 | 2.2682326 | 1.5750295 |
| 19 | 512x512 | HYPER Iterative | RS 0.77 | 2.2676442 | 1.5641388 |
| 19 | 512x512 | HYPER Iterative | RS 0.84 | 2.2664676 | 1.5537912 |
| 19 | 512x512 | HYPER Iterative | RS 0.91 | 2.2517579 | 1.5319508 |
| 19 | 512x512 | HYPER DPR | Str 2 | 2.8960419 | 2.3207904 |
| 19 | 512x512 | HYPER DPR | Str 3 | 2.9913607 | 2.4035886 |
| 19 | 512x512 | HYPER DPR | Str 4 | 3.1453588 | 2.5342713 |
| 20 | 256x256 | OSEM | Smooth 3 | 2.3812032 | 1.643041 |
| 20 | 256x256 | HYPER Iterative | RS 0.7 | 4.8971467 | 3.3141972 |
| 20 | 256x256 | HYPER Iterative | RS 0.77 | 4.8112421 | 3.2283045 |
| 20 | 256x256 | HYPER Iterative | RS 0.84 | 4.7259259 | 3.1467564 |
| 20 | 256x256 | HYPER Iterative | RS 0.91 | 4.5288162 | 2.9911165 |
| 20 | 256x256 | HYPER DPR | Str 2 | 5.6461639 | 3.9014515 |
| 20 | 256x256 | HYPER DPR | Str 3 | 5.9003468 | 4.2157242 |
| 20 | 256x256 | HYPER DPR | Str 4 | 6.1562953 | 4.5727945 |
| 20 | 512x512 | OSEM | Smooth 3 | 2.6412702 | 1.8068953 |
| 20 | 512x512 | HYPER Iterative | RS 0.7 | 6.4487238 | 4.477905 |
| 20 | 512x512 | HYPER Iterative | RS 0.77 | 6.4057713 | 4.4184689 |
| 20 | 512x512 | HYPER Iterative | RS 0.84 | 6.3592892 | 4.3596511 |
| 20 | 512x512 | HYPER Iterative | RS 0.91 | 6.1863031 | 4.2087617 |
| 20 | 512x512 | HYPER DPR | Str 2 | 9.490097 | 7.6050439 |
| 20 | 512x512 | HYPER DPR | Str 3 | 9.4924507 | 7.6272802 |
| 20 | 512x512 | HYPER DPR | Str 4 | 9.489377 | 7.6457592 |
| 21 | 256x256 | OSEM | Smooth 3 | 1.3174785 | 0.6115958 |
| 21 | 256x256 | HYPER Iterative | RS 0.7 | 1.294866 | 0.5878446 |
| 21 | 256x256 | HYPER Iterative | RS 0.77 | 1.2526162 | 0.5665474 |
| 21 | 256x256 | HYPER Iterative | RS 0.84 | 1.2145319 | 0.5477119 |
| 21 | 256x256 | HYPER Iterative | RS 0.91 | 1.1746625 | 0.5285042 |
| 21 | 256x256 | HYPER DPR | Str 2 | 1.401383 | 0.6451104 |
| 21 | 256x256 | HYPER DPR | Str 3 | 1.4995691 | 0.6936706 |
| 21 | 256x256 | HYPER DPR | Str 4 | 1.6334592 | 0.7647898 |
| 21 | 512x512 | OSEM | Smooth 3 | 1.3763901 | 0.6450709 |
| 21 | 512x512 | HYPER Iterative | RS 0.7 | 1.5947798 | 0.7458239 |
| 21 | 512x512 | HYPER Iterative | RS 0.77 | 1.534083 | 0.7145571 |
| 21 | 512x512 | HYPER Iterative | RS 0.84 | 1.4823122 | 0.6881059 |
| 21 | 512x512 | HYPER Iterative | RS 0.91 | 1.4263759 | 0.6594533 |
| 21 | 512x512 | HYPER DPR | Str 2 | 2.5843763 | 1.216848 |
| 21 | 512x512 | HYPER DPR | Str 3 | 2.7872944 | 1.320204 |
| 21 | 512x512 | HYPER DPR | Str 4 | 3.0663807 | 1.4777907 |
| 22 | 256x256 | OSEM | Smooth 3 | 1.5739524 | 0.7306553 |
| 22 | 256x256 | HYPER Iterative | RS 0.7 | 1.2924858 | 0.7385782 |
| 22 | 256x256 | HYPER Iterative | RS 0.77 | 1.2430952 | 0.707804 |
| 22 | 256x256 | HYPER Iterative | RS 0.84 | 1.2014405 | 0.6821861 |
| 22 | 256x256 | HYPER Iterative | RS 0.91 | 1.1728772 | 0.6647848 |
| 22 | 256x256 | HYPER DPR | Str 2 | 1.68523 | 0.957895 |
| 22 | 256x256 | HYPER DPR | Str 3 | 1.8328067 | 1.0579411 |
| 22 | 256x256 | HYPER DPR | Str 4 | 2.0190625 | 1.1916291 |
| 22 | 512x512 | OSEM | Smooth 3 | 1.649526 | 0.9798525 |
| 22 | 512x512 | HYPER Iterative | RS 0.7 | 1.6971313 | 0.9914425 |
| 22 | 512x512 | HYPER Iterative | RS 0.77 | 1.613227 | 0.9395295 |
| 22 | 512x512 | HYPER Iterative | RS 0.84 | 1.5406287 | 0.895011 |
| 22 | 512x512 | HYPER Iterative | RS 0.91 | 1.4632701 | 0.8482069 |
| 22 | 512x512 | HYPER DPR | Str 2 | 2.8105018 | 1.7256439 |
| 22 | 512x512 | HYPER DPR | Str 3 | 3.0669758 | 1.8844163 |
| 22 | 512x512 | HYPER DPR | Str 4 | 3.3912873 | 2.0975646 |
| 23 | 256x256 | OSEM | Smooth 3 | 1.1659119 | 0.6844077 |
| 23 | 256x256 | HYPER Iterative | RS 0.7 | 1.2801261 | 0.7315153 |
| 23 | 256x256 | HYPER Iterative | RS 0.77 | 1.2157719 | 0.6922465 |
| 23 | 256x256 | HYPER Iterative | RS 0.84 | 1.1572154 | 0.6570748 |
| 23 | 256x256 | HYPER Iterative | RS 0.91 | 1.1003982 | 0.6237038 |
| 23 | 256x256 | HYPER DPR | Str 2 | 1.4528967 | 0.8258353 |
| 23 | 256x256 | HYPER DPR | Str 3 | 1.6030564 | 0.9253236 |
| 23 | 256x256 | HYPER DPR | Str 4 | 1.8053951 | 1.0655249 |
| 23 | 512x512 | OSEM | Smooth 3 | 1.2783867 | 0.7593881 |
| 23 | 512x512 | HYPER Iterative | RS 0.7 | 2.0651307 | 1.2064231 |
| 23 | 512x512 | HYPER Iterative | RS 0.77 | 1.9271463 | 1.1223534 |
| 23 | 512x512 | HYPER Iterative | RS 0.84 | 1.8030761 | 1.0474769 |
| 23 | 512x512 | HYPER Iterative | RS 0.91 | 1.6627724 | 0.9638515 |
| 23 | 512x512 | HYPER DPR | Str 2 | 3.389899 | 2.0813929 |
| 23 | 512x512 | HYPER DPR | Str 3 | 3.603833 | 2.214273 |
| 23 | 512x512 | HYPER DPR | Str 4 | 3.9476349 | 2.4416743 |

**Abbreviations:** IMLN, internal mammary lymph node; SUV_max_, maximum standardized uptake value; TBR, tumor-to-background ratio.

*Values correspond to the quantitative data visualized in Figure 5.

*Lesion numbering follows the same order as in Figure 5.

**Supplementary Table S2. Patient-based background noise (CV_BG_) on clinical PET/CT images**

| Patient. No. | Matrix size | Reconstruction | Parameter | CV_BG_ |
| --- | --- | --- | --- | --- |
| 1 | 256x256 | OSEM | Smooth 3 | 0.096267 |
| 1 | 256x256 | HYPER Iterative | RS 0.7 | 0.106346 |
| 1 | 256x256 | HYPER Iterative | RS 0.77 | 0.099845 |
| 1 | 256x256 | HYPER Iterative | RS 0.84 | 0.094078 |
| 1 | 256x256 | HYPER Iterative | RS 0.91 | 0.088556 |
| 1 | 256x256 | HYPER DPR | Str 2 | 0.072826 |
| 1 | 256x256 | HYPER DPR | Str 3 | 0.096942 |
| 1 | 256x256 | HYPER DPR | Str 4 | 0.124003 |
| 1 | 512x512 | OSEM | Smooth 3 | 0.114828 |
| 1 | 512x512 | HYPER Iterative | RS 0.7 | 0.188198 |
| 1 | 512x512 | HYPER Iterative | RS 0.77 | 0.175134 |
| 1 | 512x512 | HYPER Iterative | RS 0.84 | 0.163744 |
| 1 | 512x512 | HYPER Iterative | RS 0.91 | 0.151953 |
| 1 | 512x512 | HYPER DPR | Str 2 | 0.263649 |
| 1 | 512x512 | HYPER DPR | Str 3 | 0.305158 |
| 1 | 512x512 | HYPER DPR | Str 4 | 0.364237 |
| 2 | 256x256 | OSEM | Smooth 3 | 0.057687 |
| 2 | 256x256 | HYPER Iterative | RS 0.7 | 0.059761 |
| 2 | 256x256 | HYPER Iterative | RS 0.77 | 0.055193 |
| 2 | 256x256 | HYPER Iterative | RS 0.84 | 0.051477 |
| 2 | 256x256 | HYPER Iterative | RS 0.91 | 0.047362 |
| 2 | 256x256 | HYPER DPR | Str 2 | 0.048407 |
| 2 | 256x256 | HYPER DPR | Str 3 | 0.066151 |
| 2 | 256x256 | HYPER DPR | Str 4 | 0.093296 |
| 2 | 512x512 | OSEM | Smooth 3 | 0.068256 |
| 2 | 512x512 | HYPER Iterative | RS 0.7 | 0.120921 |
| 2 | 512x512 | HYPER Iterative | RS 0.77 | 0.110184 |
| 2 | 512x512 | HYPER Iterative | RS 0.84 | 0.10124 |
| 2 | 512x512 | HYPER Iterative | RS 0.91 | 0.092064 |
| 2 | 512x512 | HYPER DPR | Str 2 | 0.218643 |
| 2 | 512x512 | HYPER DPR | Str 3 | 0.247687 |
| 2 | 512x512 | HYPER DPR | Str 4 | 0.293503 |
| 3 | 256x256 | OSEM | Smooth 3 | 0.109509 |
| 3 | 256x256 | HYPER Iterative | RS 0.7 | 0.091189 |
| 3 | 256x256 | HYPER Iterative | RS 0.77 | 0.083206 |
| 3 | 256x256 | HYPER Iterative | RS 0.84 | 0.076328 |
| 3 | 256x256 | HYPER Iterative | RS 0.91 | 0.069366 |
| 3 | 256x256 | HYPER DPR | Str 2 | 0.088381 |
| 3 | 256x256 | HYPER DPR | Str 3 | 0.113411 |
| 3 | 256x256 | HYPER DPR | Str 4 | 0.153982 |
| 3 | 512x512 | OSEM | Smooth 3 | 0.135584 |
| 3 | 512x512 | HYPER Iterative | RS 0.7 | 0.167434 |
| 3 | 512x512 | HYPER Iterative | RS 0.77 | 0.153632 |
| 3 | 512x512 | HYPER Iterative | RS 0.84 | 0.141734 |
| 3 | 512x512 | HYPER Iterative | RS 0.91 | 0.128678 |
| 3 | 512x512 | HYPER DPR | Str 2 | 0.324958 |
| 3 | 512x512 | HYPER DPR | Str 3 | 0.357285 |
| 3 | 512x512 | HYPER DPR | Str 4 | 0.409474 |
| 4 | 256x256 | OSEM | Smooth 3 | 0.083757 |
| 4 | 256x256 | HYPER Iterative | RS 0.7 | 0.094151 |
| 4 | 256x256 | HYPER Iterative | RS 0.77 | 0.089445 |
| 4 | 256x256 | HYPER Iterative | RS 0.84 | 0.085655 |
| 4 | 256x256 | HYPER Iterative | RS 0.91 | 0.082214 |
| 4 | 256x256 | HYPER DPR | Str 2 | 0.075143 |
| 4 | 256x256 | HYPER DPR | Str 3 | 0.095843 |
| 4 | 256x256 | HYPER DPR | Str 4 | 0.131082 |
| 4 | 512x512 | OSEM | Smooth 3 | 0.103967 |
| 4 | 512x512 | HYPER Iterative | RS 0.7 | 0.158173 |
| 4 | 512x512 | HYPER Iterative | RS 0.77 | 0.14843 |
| 4 | 512x512 | HYPER Iterative | RS 0.84 | 0.140339 |
| 4 | 512x512 | HYPER Iterative | RS 0.91 | 0.132244 |
| 4 | 512x512 | HYPER DPR | Str 2 | 0.240721 |
| 4 | 512x512 | HYPER DPR | Str 3 | 0.271399 |
| 4 | 512x512 | HYPER DPR | Str 4 | 0.322551 |
| 5 | 256x256 | OSEM | Smooth 3 | 0.070461 |
| 5 | 256x256 | HYPER Iterative | RS 0.7 | 0.068587 |
| 5 | 256x256 | HYPER Iterative | RS 0.77 | 0.062722 |
| 5 | 256x256 | HYPER Iterative | RS 0.84 | 0.057929 |
| 5 | 256x256 | HYPER Iterative | RS 0.91 | 0.053346 |
| 5 | 256x256 | HYPER DPR | Str 2 | 0.06347 |
| 5 | 256x256 | HYPER DPR | Str 3 | 0.084438 |
| 5 | 256x256 | HYPER DPR | Str 4 | 0.11347 |
| 5 | 512x512 | OSEM | Smooth 3 | 0.088034 |
| 5 | 512x512 | HYPER Iterative | RS 0.7 | 0.145686 |
| 5 | 512x512 | HYPER Iterative | RS 0.77 | 0.132472 |
| 5 | 512x512 | HYPER Iterative | RS 0.84 | 0.121329 |
| 5 | 512x512 | HYPER Iterative | RS 0.91 | 0.110048 |
| 5 | 512x512 | HYPER DPR | Str 2 | 0.247857 |
| 5 | 512x512 | HYPER DPR | Str 3 | 0.284218 |
| 5 | 512x512 | HYPER DPR | Str 4 | 0.342656 |
| 6 | 256x256 | OSEM | Smooth 3 | 0.070626 |
| 6 | 256x256 | HYPER Iterative | RS 0.7 | 0.06781 |
| 6 | 256x256 | HYPER Iterative | RS 0.77 | 0.061858 |
| 6 | 256x256 | HYPER Iterative | RS 0.84 | 0.056783 |
| 6 | 256x256 | HYPER Iterative | RS 0.91 | 0.051594 |
| 6 | 256x256 | HYPER DPR | Str 2 | 0.069459 |
| 6 | 256x256 | HYPER DPR | Str 3 | 0.089134 |
| 6 | 256x256 | HYPER DPR | Str 4 | 0.123937 |
| 6 | 512x512 | OSEM | Smooth 3 | 0.092026 |
| 6 | 512x512 | HYPER Iterative | RS 0.7 | 0.135415 |
| 6 | 512x512 | HYPER Iterative | RS 0.77 | 0.12392 |
| 6 | 512x512 | HYPER Iterative | RS 0.84 | 0.113999 |
| 6 | 512x512 | HYPER Iterative | RS 0.91 | 0.10351 |
| 6 | 512x512 | HYPER DPR | Str 2 | 0.239096 |
| 6 | 512x512 | HYPER DPR | Str 3 | 0.266915 |
| 6 | 512x512 | HYPER DPR | Str 4 | 0.314422 |
| 7 | 256x256 | OSEM | Smooth 3 | 0.090737 |
| 7 | 256x256 | HYPER Iterative | RS 0.7 | 0.074573 |
| 7 | 256x256 | HYPER Iterative | RS 0.77 | 0.068255 |
| 7 | 256x256 | HYPER Iterative | RS 0.84 | 0.062975 |
| 7 | 256x256 | HYPER Iterative | RS 0.91 | 0.058222 |
| 7 | 256x256 | HYPER DPR | Str 2 | 0.070392 |
| 7 | 256x256 | HYPER DPR | Str 3 | 0.09296 |
| 7 | 256x256 | HYPER DPR | Str 4 | 0.12781 |
| 7 | 512x512 | OSEM | Smooth 3 | 0.100805 |
| 7 | 512x512 | HYPER Iterative | RS 0.7 | 0.140128 |
| 7 | 512x512 | HYPER Iterative | RS 0.77 | 0.12725 |
| 7 | 512x512 | HYPER Iterative | RS 0.84 | 0.116264 |
| 7 | 512x512 | HYPER Iterative | RS 0.91 | 0.105625 |
| 7 | 512x512 | HYPER DPR | Str 2 | 0.276905 |
| 7 | 512x512 | HYPER DPR | Str 3 | 0.306052 |
| 7 | 512x512 | HYPER DPR | Str 4 | 0.355088 |
| 8 | 256x256 | OSEM | Smooth 3 | 0.075337 |
| 8 | 256x256 | HYPER Iterative | RS 0.7 | 0.086701 |
| 8 | 256x256 | HYPER Iterative | RS 0.77 | 0.077913 |
| 8 | 256x256 | HYPER Iterative | RS 0.84 | 0.070416 |
| 8 | 256x256 | HYPER Iterative | RS 0.91 | 0.063537 |
| 8 | 256x256 | HYPER DPR | Str 2 | 0.102309 |
| 8 | 256x256 | HYPER DPR | Str 3 | 0.128625 |
| 8 | 256x256 | HYPER DPR | Str 4 | 0.168096 |
| 8 | 512x512 | OSEM | Smooth 3 | 0.094673 |
| 8 | 512x512 | HYPER Iterative | RS 0.7 | 0.171616 |
| 8 | 512x512 | HYPER Iterative | RS 0.77 | 0.156668 |
| 8 | 512x512 | HYPER Iterative | RS 0.84 | 0.143581 |
| 8 | 512x512 | HYPER Iterative | RS 0.91 | 0.129804 |
| 8 | 512x512 | HYPER DPR | Str 2 | 0.503393 |
| 8 | 512x512 | HYPER DPR | Str 3 | 0.522901 |
| 8 | 512x512 | HYPER DPR | Str 4 | 0.556726 |
| 9 | 256x256 | OSEM | Smooth 3 | 0.058507 |
| 9 | 256x256 | HYPER Iterative | RS 0.7 | 0.04425 |
| 9 | 256x256 | HYPER Iterative | RS 0.77 | 0.040743 |
| 9 | 256x256 | HYPER Iterative | RS 0.84 | 0.037768 |
| 9 | 256x256 | HYPER Iterative | RS 0.91 | 0.035012 |
| 9 | 256x256 | HYPER DPR | Str 2 | 0.044326 |
| 9 | 256x256 | HYPER DPR | Str 3 | 0.055164 |
| 9 | 256x256 | HYPER DPR | Str 4 | 0.076518 |
| 9 | 512x512 | OSEM | Smooth 3 | 0.063641 |
| 9 | 512x512 | HYPER Iterative | RS 0.7 | 0.099485 |
| 9 | 512x512 | HYPER Iterative | RS 0.77 | 0.090086 |
| 9 | 512x512 | HYPER Iterative | RS 0.84 | 0.082123 |
| 9 | 512x512 | HYPER Iterative | RS 0.91 | 0.074345 |
| 9 | 512x512 | HYPER DPR | Str 2 | 0.188097 |
| 9 | 512x512 | HYPER DPR | Str 3 | 0.215003 |
| 9 | 512x512 | HYPER DPR | Str 4 | 0.258612 |
| 10 | 256x256 | OSEM | Smooth 3 | 0.090435 |
| 10 | 256x256 | HYPER Iterative | RS 0.7 | 0.071793 |
| 10 | 256x256 | HYPER Iterative | RS 0.77 | 0.065407 |
| 10 | 256x256 | HYPER Iterative | RS 0.84 | 0.060049 |
| 10 | 256x256 | HYPER Iterative | RS 0.91 | 0.054802 |
| 10 | 256x256 | HYPER DPR | Str 2 | 0.067495 |
| 10 | 256x256 | HYPER DPR | Str 3 | 0.084278 |
| 10 | 256x256 | HYPER DPR | Str 4 | 0.113408 |
| 10 | 512x512 | OSEM | Smooth 3 | 0.108962 |
| 10 | 512x512 | HYPER Iterative | RS 0.7 | 0.139394 |
| 10 | 512x512 | HYPER Iterative | RS 0.77 | 0.12721 |
| 10 | 512x512 | HYPER Iterative | RS 0.84 | 0.116739 |
| 10 | 512x512 | HYPER Iterative | RS 0.91 | 0.105713 |
| 10 | 512x512 | HYPER DPR | Str 2 | 0.269765 |
| 10 | 512x512 | HYPER DPR | Str 3 | 0.297976 |
| 10 | 512x512 | HYPER DPR | Str 4 | 0.345164 |
| 11 | 256x256 | OSEM | Smooth 3 | 0.122638 |
| 11 | 256x256 | HYPER Iterative | RS 0.7 | 0.105694 |
| 11 | 256x256 | HYPER Iterative | RS 0.77 | 0.100189 |
| 11 | 256x256 | HYPER Iterative | RS 0.84 | 0.095313 |
| 11 | 256x256 | HYPER Iterative | RS 0.91 | 0.089791 |
| 11 | 256x256 | HYPER DPR | Str 2 | 0.122312 |
| 11 | 256x256 | HYPER DPR | Str 3 | 0.141769 |
| 11 | 256x256 | HYPER DPR | Str 4 | 0.172863 |
| 11 | 512x512 | OSEM | Smooth 3 | 0.133555 |
| 11 | 512x512 | HYPER Iterative | RS 0.7 | 0.161156 |
| 11 | 512x512 | HYPER Iterative | RS 0.77 | 0.149727 |
| 11 | 512x512 | HYPER Iterative | RS 0.84 | 0.140479 |
| 11 | 512x512 | HYPER Iterative | RS 0.91 | 0.131429 |
| 11 | 512x512 | HYPER DPR | Str 2 | 0.339397 |
| 11 | 512x512 | HYPER DPR | Str 3 | 0.375362 |
| 11 | 512x512 | HYPER DPR | Str 4 | 0.429762 |
| 12 | 256x256 | OSEM | Smooth 3 | 0.072675 |
| 12 | 256x256 | HYPER Iterative | RS 0.7 | 0.060179 |
| 12 | 256x256 | HYPER Iterative | RS 0.77 | 0.054035 |
| 12 | 256x256 | HYPER Iterative | RS 0.84 | 0.048849 |
| 12 | 256x256 | HYPER Iterative | RS 0.91 | 0.043991 |
| 12 | 256x256 | HYPER DPR | Str 2 | 0.061542 |
| 12 | 256x256 | HYPER DPR | Str 3 | 0.077236 |
| 12 | 256x256 | HYPER DPR | Str 4 | 0.100525 |
| 12 | 512x512 | OSEM | Smooth 3 | 0.091101 |
| 12 | 512x512 | HYPER Iterative | RS 0.7 | 0.139464 |
| 12 | 512x512 | HYPER Iterative | RS 0.77 | 0.126641 |
| 12 | 512x512 | HYPER Iterative | RS 0.84 | 0.115899 |
| 12 | 512x512 | HYPER Iterative | RS 0.91 | 0.104803 |
| 12 | 512x512 | HYPER DPR | Str 2 | 0.246487 |
| 12 | 512x512 | HYPER DPR | Str 3 | 0.2802 |
| 12 | 512x512 | HYPER DPR | Str 4 | 0.338642 |
| 13 | 256x256 | OSEM | Smooth 3 | 0.095745 |
| 13 | 256x256 | HYPER Iterative | RS 0.7 | 0.107347 |
| 13 | 256x256 | HYPER Iterative | RS 0.77 | 0.098869 |
| 13 | 256x256 | HYPER Iterative | RS 0.84 | 0.091203 |
| 13 | 256x256 | HYPER Iterative | RS 0.91 | 0.083836 |
| 13 | 256x256 | HYPER DPR | Str 2 | 0.085579 |
| 13 | 256x256 | HYPER DPR | Str 3 | 0.109316 |
| 13 | 256x256 | HYPER DPR | Str 4 | 0.140844 |
| 13 | 512x512 | OSEM | Smooth 3 | 0.100318 |
| 13 | 512x512 | HYPER Iterative | RS 0.7 | 0.167839 |
| 13 | 512x512 | HYPER Iterative | RS 0.77 | 0.156295 |
| 13 | 512x512 | HYPER Iterative | RS 0.84 | 0.146015 |
| 13 | 512x512 | HYPER Iterative | RS 0.91 | 0.13555 |
| 13 | 512x512 | HYPER DPR | Str 2 | 0.268297 |
| 13 | 512x512 | HYPER DPR | Str 3 | 0.292039 |
| 13 | 512x512 | HYPER DPR | Str 4 | 0.330394 |
